# Annellated 1,3,4,2-Triazaphospholenes-Simple Modular Synthesis and a First Exploration of Ligand Properties

**DOI:** 10.3390/molecules27154747

**Published:** 2022-07-25

**Authors:** Ferdinand Richter, Nicholas Birchall, Christoph M. Feil, Martin Nieger, Dietrich Gudat

**Affiliations:** 1 Institut für Anorganische Chemie, University of Stuttgart, 70550 Stuttgart, Germany; ferdinand.richter@iac.uni-stuttgart.de (F.R.); nicholas.birchall@iac.uni-stuttgart.de (N.B.); christoph.feil@gmx.de (C.M.F.); 2 Department of Chemistry, University of Helsinki, FIN-00014 Helsinki, Finland; martin.nieger@helsinki.fi

**Keywords:** phosphorus nitrogen heterocycles, [1 + 4]-cycloaddition, N-heterocyclic phosphines, phosphine complexes, P-donor ligands

## Abstract

The successful use of 1,3,4,2-triazaphospholenes (TAPs) as organo-catalysts stresses the need for efficient synthetic routes to these molecules. In this study, we establish the [1 + 4]-cycloaddition of PBr_3_ to azo-pyridines as a new approach to preparing pyrido-annellated TAPs in a single step from easily available precursors. The modular assembly of the azo-component via condensation of primary amines and nitroso compounds along with the feasibility of post-functionalization at the P–Br bond under conservation of the heterocyclic structure allows, in principle, to address a wide range of target molecules, which is illustrated by prototypical examples. The successful synthesis of a transition metal complex confirms for the first time the ability of a TAP to act as a P-donor ligand. Crystallographic studies suggest that hyperconjugation effects and intermolecular interactions induce a qualitatively similar ionic polarization of the P–Br bonds in TAPs as in better known isoelectronic diazaphospholenes.

## 1. Introduction

**Scheme 1:**
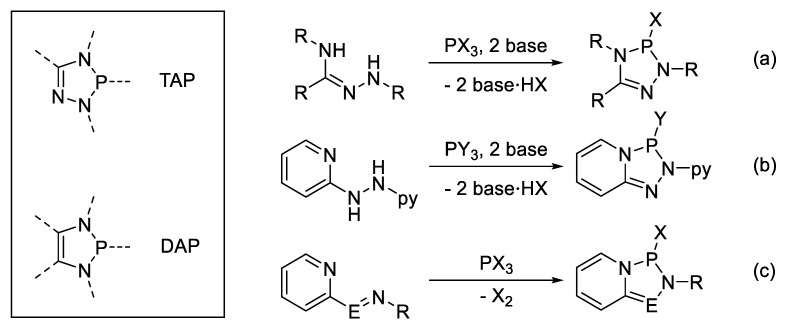
Generic molecular structures of 1,3,4,2-triazaphospholenes (TAPs) and 1,3,2-diazaphospholenes (DAPs), synthetic routes to TAPs involving base-induced condensations of phosphorus trihalides with (**a**) amidrazones and (**b**) hydrazobispyridine, and (**c**) assembly of heterocycles via cycloaddition of phosphorus trihalides to imino-(E = CH) and azo-pyridines (E = N), respectively. (R = organic substituent; X = halogen; Y = Cl, NEt_2_; py = 2-pyridin-yl).

The 1,3,4,2-triazaphospholenes (TAPs, Scheme 1) are isoelectronic analogues of the better-known 1,3,2-diazaphospholenes (DAPs) [1,2,3] which have gathered some attention as broadly applicable organo-catalysts [4,5,6]. The synthesis of TAPs is traditionally accomplished via the base-induced condensation of amidrazones with PCl_3_ (Scheme 1a) [7], and a specimen obtained via this route has likewise been shown to exhibit catalytic activity in the hydroboration of imines and α, β-unsaturated aldehydes [8]. An advantage of using TAPs as (pre)catalysts was claimed to be that the easy assembly of target compounds with unlike N-substituents promises to overcome a drawback of all currently known approaches to DAPs, which do not permit a general synthesis of non-symmetric phosphorus heterocycles containing smaller alkyl groups [5]. The TAP framework can thus be considered a more modular synthetic scaffold. This increased flexibility facilitating, in principle, the tailoring of molecular structures, e.g., for the optimization of a catalytic process, further development of the synthetic chemistry of TAPs would seem highly desirable.

Recently, the range of unsymmetrically substituted TAPs was extended by the synthesis of pyrido-annellated derivatives (2,3-dihydro-[1,2,4,3] triazaphospholo [4,5-a] pyridines) via base-induced condensation of hydrazo-bis-pyridine with PCl_3_ or (Et_2_N)_2_PCl, respectively (Scheme 1b) [9]. Although post-modification via formal exchange of a P-amino-substituent for halides was feasible, the scope of this approach is still limited by the synthesis of target molecules carrying a 2-pyridin-2-yl substituent. Moreover, the one-step preparation of the synthetically valuable P-chloro-derivative is hampered by its low solubility in common organic solvents as well as difficulties in its separation from hydrochloride salts formed as by-products.

We had previously shown that pyrido-annellated DAPs form readily under salt-free conditions via a [4 + 1]-cycloaddition of PI_3_ to imino-pyridines (Scheme 1c, E = CH) [10]. Considering that this reaction is just a special case of a more general access route to DAPs via cycloaddition of phosphorus trihalides to diimines [11,12], we anticipated that extending this scheme to azopyridines should furnish a direct and practically viable new route to pyrido-annellated TAPs (Scheme 1c, E = N). Here, we report on the elaboration of this concept into a modular synthesis of TAPs and reveal that P-halogeno-TAPs are amenable to P-alkylation under the retention of the heterocyclic framework. The synthetic procedures described open a new and highly efficient route to the assembly of a wider range of TAPs with non-symmetrical molecular structures. Moreover, a preliminary exploration of the complexation properties of the P-alkylated product resulting in the isolation of a first complex comprising a P-coordinated TAP as ligand should stimulate further studies of the use of these heterocycles in coordination chemistry and catalysis.

## 2. Results and Discussion

### 2.1. Synthesis of TAPs

**Scheme 2:**
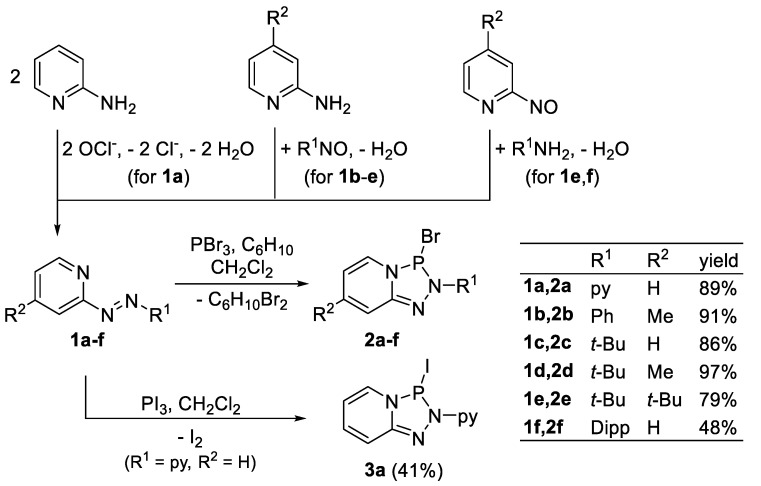
Synthesis of TAPs from phosphorus trihalides and azopyridines (py = 2-pyridin-yl). Yields refer to isolated yields of **2a**–**f**.

The reactions studied for the assembly of the TAP ring include the [1 + 4]-cycloadditions of both symmetrical (**1a**) and unsymmetrical (**1b**–**f**) azopyridines with PBr_3_ and PI_3_, respectively (Scheme 2). Most azo-components employed were conveniently prepared from readily available aminopyridines using established protocols to accomplish either the oxidative coupling of two molecules of parent aminopyridine to afford **1a** [13], or the condensation of an aminopyridine with an appropriate nitroso-alkane or nitroso-arene [14,15] to furnish **1b**–**e**, respectively. The preparation of 1e was also feasible in an even higher yield through a complementary condensation of nitroso-pyridine with *t*-butylamine, and **1f** was exclusively accessed via this route. The choice of the phosphorus source was stimulated by our previous mechanistic studies on the formation of DAPs via the cycloaddition route [16], which allowed us to establish that reactions of diimines with PBr_3_ or PI_3_ proceed smoothly to the desired heterocycles while the corresponding reaction with PCl_3_ takes a different course.

Actual ring closure reactions of azopyridines with PBr_3_ were carried out, by analogy to an established protocol for the synthesis of DAPs [12], using dichloromethane as a solvent and a stoichiometric amount of cyclohexene to scavenge elementary bromine formed as co-product. Standard work-up procedures furnished acceptable to excellent yields (48–97%) of TAPs **2a**–**f** in the form of yellow, moisture-sensitive solids. The identity and purity of all products were established by analytical and spectroscopic data, and in several cases by single-crystal XRD studies (vide infra). Compound **2a** had previously been prepared in two steps and lower overall yield from PBr_3_ and hydrazobispyridine [9]. The presence of a varying number of alkyl groups rendered **2c**–**f** more soluble in organic solvents than **2a**,**b** as expected.

Analogous reactions of selected azopyridines with PI_3_ were—like the corresponding syntheses of DAPs [11]—carried out without an additional halogen-scavenger. While ^31^P NMR spectroscopic reaction monitoring gave evidence that heterocycle formation had occurred as expected, work-up was in this case compromised by the formation of dynamic equilibrium mixtures containing ionic triazaphospholenium triiodides along with P-iodo-TAPs and I_2_, as well as a high liability of the products to undergo hydrolysis and further unclear dismutation reactions. Such complications impeding in most cases the isolation of analytically pure products, we found these transformations generally of little synthetic use. A notable exception from this scheme was only the reaction of PI_3_ with azobispyridine **1a**, which produced a crop of red, crystalline material identified as iodo-TAP **3a** (Scheme 2). While the molecular structure of **3a** seems at first atypical in view of the fact that analogous cycloadditions of PI_3_ with diimines and imino-pyridines yield isolable ionic diazaphospholenium triiodes rather than simple P-iodo DAPs [10,11], the discrepancy is easily rationalized in terms of different Lewis acidities of the cationic triaza- and diazaphospholenium units generated by abstraction of iodide from a P-iodo-TAP or DAP, respectively: the formal replacement of a CH-fragment in a diazaphospholenium ion by an isoelectronic nitrogen atom making the resulting triazaphospholenium ion a stronger Lewis-acid, it also renders release of an iodide from a neutral TAP energetically less favorable than from a DAP, and may thus inhibit triiodide formation by shifting the equilibrium >P–I + I_2_ ⇆ >P^+^ I_3_^–^ further to the left side. We presume that the interplay of this effect with different solubilities of the equilibrating species is also well suited to explain the complications encountered during the work-up of reaction mixtures (vide supra).

We want to note that the observed reactivity of 1a towards PBr_3_ and PI_3_ contrasts its behavior toward PCl_3_, which had previously been reported to react under similar conditions to afford no TAP but rather an elusive 1:1 adduct that remained unidentified and was only chemically characterized by its conversion into a mixture of hydrazobispyridine and trimethylphosphate upon methanolysis [9]. Interestingly, we had noted earlier that a similar disparity occurs as well in the reactions of diimines with PCl_3_ and its heavier congeners [16]. Computational studies suggested that while the formation of P-halogeno-DAPs via an oxidative addition/reductive elimination sequence ([1 + 4]-cycloaddition of PX_3_ to the diimine followed by cleavage of X_2_) is in this case in principle feasible for all three phosphorus trihalides, the second step is energetically disfavored when the X_2_ molecule released is a strong oxidant. As a result, the formation of DAPs from PX_3_ turns from a thermodynamically favored exergonic reaction for X = Br, I into a strongly endergonic process that can no longer compete with alternative reaction channels for X = Cl [16]. Presuming that the assembly of TAPs from azopyridines and PBr_3_ or PI_3_ occurs likewise via an oxidative addition/reductive elimination pathway and that the reported chemical behavior of the 1:1 adduct of **1a** and PCl_3_ at the same time agrees well with the presence of a [1 + 4]-cycloaddition product showing no tendency to lose Cl_2_, we relate the different reactivities of PCl_3_ and its heavier congeners towards azopyridines to the same origin.

### 2.2. Alkylation and Complexation of a TAP

**Scheme 3:**
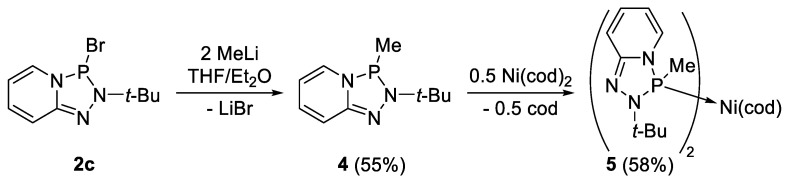
Synthesis of a P-alkylated TAP and its nickel complex (cod = 1,5-cyclooctadiene).

Anticipating that metathesis reactions at the P-halogen bonds of **2a**–**f** should open a modular route to the decoration of the TAP scaffold with organic substituents, we studied the methylation of **2c** as an archetypal example. Reaction monitoring by ^31^P NMR spectroscopy revealed that treatment with an equimolar amount of MeLi resulted in the selective formation of a product that was isolated in an acceptable yield of 55% after distillation and identified by analytical and spectroscopic data as well as a single-crystal XRD study as the expected N-heterocyclic phosphine 4 (Scheme 3). We regard the formation of 4 as confirmation that substitution at the P-halogen functionality of a TAP by organic nucleophiles is in principle feasible without disrupting the heterocyclic framework.

The presence of an electron-releasing P-alkyl substituent further triggered the expectation that the phosphorus atom in **4** might become sufficiently nucleophilic for binding to a transition metal center. Since knowledge of coordination compounds comprising TAP ligands has so far been limited to a single example featuring a 2-pyridyl-substituted molecule acting as κ^2^N,N′-donor to a hard silicon (IV) center [9], we set out to verify the validity of our hypothesis. Studying the reaction of 4 with bis-cyclooctadiene-nickel(0), we observed formation of two new products later identified as the diastereomeric complexes *rac-/meso-*5, respectively. A mixture of both diastereomers was isolated by crystallization and the expected presence of a complex containing two κ^1^P-bound TAP units confirmed by spectroscopic data and a single-crystal XRD study of the *rac*-isomer.

### 2.3. Compound Characterization

The ^1^H and ^13^C NMR data of the newly prepared TAPs confirm the expected molecular structures but reveal otherwise no special features. The ^31^P NMR chemical shifts of P-bromo-(2a-f, δ^31^P 135–156 ppm) and P-iodo-derivatives (3a, δ^31^P 173 ppm) are compatible with the presence of N_2_X-substituted phosphine derivatives but are consistently smaller than those of DAPs (δ^31^P 194–185 ppm for P–Br- and 205–190 ppm for P–I-derivatives [1]). Considering that the high ^31^P chemical shifts of P-halogeno-DAPs seem to be intimately connected with an exceptional ionic polarization and a lengthening of the P–X bonds (X = Cl, Br, I) which led to their depiction as dative donor/acceptor interactions [2,3], the values of δ^31^P for **2a**–**f** indicate that the P–X-bonds are again closer to a ‘normal’ covalent interaction. This trend is in line with the perception of the cationic triazaphospholenium unit being a stronger Lewis acid than a nitrogen-poorer diazaphospholenium cation.

**Figure 1:**
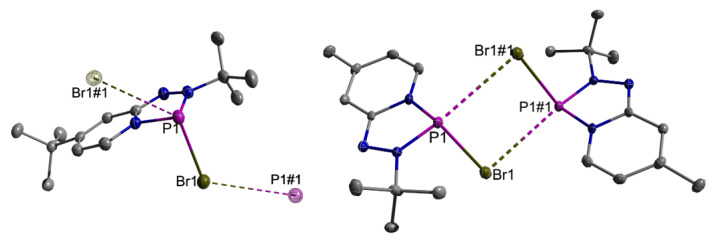
Representation of the molecular structures of **2e** (left, with contacts to the phosphorus and bromine atoms of adjacent molecules) and a dimeric unit of **2d** (right) in the crystal. Hydrogen atoms were omitted for clarity and intermolecular contacts were drawn as dashed lines. Thermal ellipsoids were drawn at the 50% probability level. Intermolecular distances (in Å): P1⋯Br1#1 3.6133 (16) (**2d**), 3.8782(6) (**2e**). Representations of the molecular structures of **2c**,**f**, and **3a** are included in the Supplementary Material, and selected distances for all compounds are listed in Table 1 and Table S2.

The single-crystal XRD studies revealed that crystals of all P–Br/I-substituted TAPs studied comprise clearly identifiable molecular units (see Figure 1). The carbon and nitrogen atoms in the fused heterocycles are generally arranged in a common plane. The phosphorus atoms in **2c**,**e**,**f**, and **3a** are slightly (by 0.13–0.23 Å) dislocated out of this plane, and the five-membered rings adopt thus a flat envelope conformation in which the P–Br bond assumes the flagpole position. In the case of **2d**, the phosphorus atom is located in the same plane as the carbon and nitrogen atoms, and the five-membered ring is planar. The six-membered rings stay planar in all compounds. Each phosphorus atom in **2a**–**f** further exhibits a single intermolecular contact with the bromine atom of a neighboring molecule, which is for **2c**–**e** below the sum of van-der-Waals radii (3.76 Å [17]). These Br^⋯^P interactions connect individual molecules to supramolecular aggregates in the form of one-dimensional infinite stacks (**2c**,**e**) or discrete dimeric units (**2d**,**f**), respectively (Figure 1).

**Figure 2:**
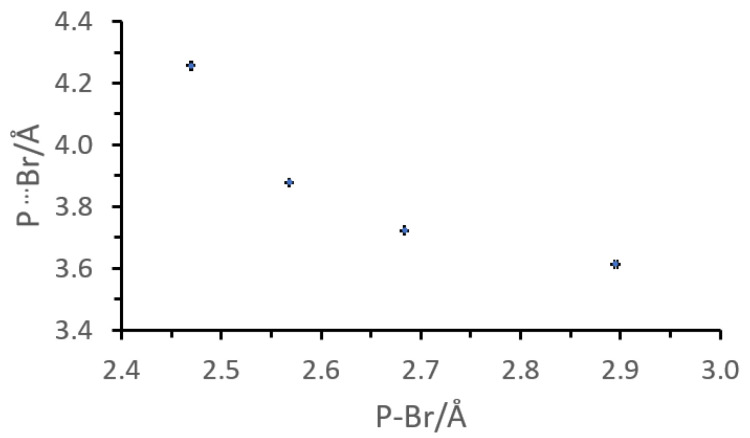
Plot of intramolecular (P–Br) vs. intermolecular (P⋯Br) distances in crystalline **2c**–**f**.

The perimeters of the fused ring systems in all bromo/iodo-TAPs are characterized by an alternating sequence of shorter and longer bonds with partly averaged distances (compared to isolated single and double bonds [18]) that is typical for conjugated π-systems in general and occurs likewise in pyrido-annellated DAPs [10]. The P–X distances in **2c**–**f** (Table 1) are shorter than in DAPs (P–Br 2.62–2.94 Å, P–I 3.43 Å [2]), but certainly exceed standard values (P–Br 2.39 Å, P–I 2.58 Å [19]) and display a comparable variability as in the nitrogen-poorer congeners. Remarkably, the trend in P–Br distances for **2c**–**f** bears no obvious relation with intramolecular substituent effects but shows a striking correlation with the aforementioned intermolecular P⋯Br contacts (Figure 2).

The observed structural trends suggest that the P–X bond lengthening in P-halogeno-TAPs arises like in the DAP system from a combination of intra- and intermolecular effects [2]. The former can be pictured as the familiar n(N)–σ*(P–X) hyperconjugation [1,2,3], which tends to increase covalent P–N bond orders relative to standard single bonds and enables π-delocalization in the annellated rings at the expense of a reduced covalency of the exocyclic P–X (X = Br, I) bonds. Intermolecular effects may be associated with weak P⋯X donor/acceptor interactions between adjacent molecules [20]. Judging by the resulting P–X distances, we may assume that, in line with the conclusions drawn from the interpretation of NMR data, the overall ionic polarization of the P–X bonds reflecting the impact of both influences is in the annellated TAPs smaller than in isoelectronic DAPs.

**Figure 3:**
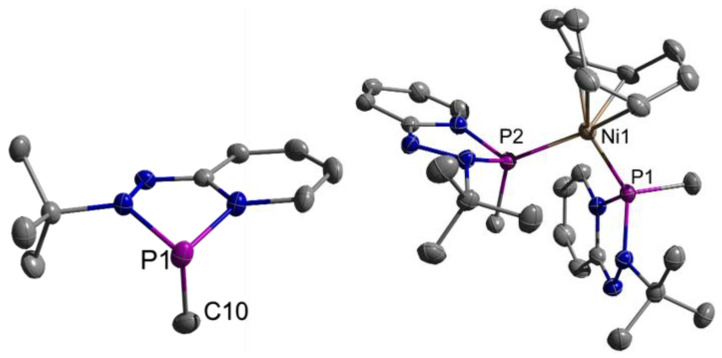
Representation of the molecular structures of 4 (**left**) and 5 (**right**) in the crystal. Hydrogen atoms were omitted for clarity. Thermal ellipsoids were drawn at the 50% probability level. Selected distances are listed in Table 1 and Table S2.

The annellated ring system in 4 (Figure 3, left) shows a similar disposition as in **2c**,**e**,**f**, and **3a**, but the folding of the five-membered ring is still more pronounced. The P–N distances are longer and may be described as normal single bonds, and a perceptible lengthening of the N–N-bond (see Table 1) epitomizes that the equalization between formal single and double bonds induced by π-delocalization effects in the remaining perimeter of the annellated rings is reduced. The local environment around the phosphorus atom seems archetypal for a trivalent phosphine and is not significantly perturbed by hyperconjugation or ionic bond polarization effects.

The geometrical features do not change significantly upon coordination of ligand 4 to a nickel center in 5 (Figure 3, right). The P–Ni distances range close to the lower end of typical values in nickel phosphine complexes (2.186 ± 0.052 Å [21]) and may be seen as an indication of rather strong metal-ligand bonding. It should be noted that the achiral crystal (point group *P*-1) contains exclusively the enantiomers [(*R*-4)_2_Ni(COD)] and [(*S*-4)_2_Ni(COD)] of a homochiral complex (*rac*-5), while the bulk sample is a 1:1 mixture of *rac*-5 and its heterochiral diastereomer [(*R*-4)(*S*-4)Ni(COD)] (*meso*-5). This finding points to the existence of a second crystalline phase containing the other diastereomer of **5**, which so far evaded crystallographic characterization.

## 3. Materials and Methods

All manipulations were carried out under an atmosphere of inert argon inside glove boxes or by using standard vacuum line techniques. Solvents were dried by literature known procedures [22] and distilled before use. NMR spectra were recorded on Bruker Avance 250 (^1^H 250.0 MHz, ^13^C 62.9 MHz, ^31^P 101.2 MHz) or Avance 400 (^1^H 400.1 MHz, ^13^C 100.5 MHz, ^31^P 161.9 MHz) instruments at 293 K if not stated otherwise. ^1^H Chemical shifts were referenced to TMS using the signals of the residual protons of the deuterated solvent (δ^1^H = 7.15 (C_6_D_6_), 1.73 (THF-D_8_)) as secondary reference. Atoms in pyridine rings were denoted as H-6, 6-C etc. Spectra of heteronuclei were referenced using the Ξ-scale [23] employing TMS (Ξ = 25.145020 MHz, ^13^C) and 85 % H_3_PO_4_ (Ξ = 40.480747 MHz, ^31^P) as secondary references. Elemental analyses were determined on a Thermo Micro Cube CHN analyzer. Previously known azopyridines were prepared as reported [9,14] (data for 1b: yield 58%; ^1^H NMR (CDCl_3_): δ = 2.39 (br s, 3 H, CH_3_), 7.15 (dq, ^3^*J*_HH_ = 4.9 Hz, ^4^*J*_HH_ = 0.8 Hz, 1 H, H-5), 7.42–7.50 (m, 3 H, *m*/*p*-Ph), 7.57 (m, 1 H, H-3), 7.95 (m, 2 H, *o*-Ph), 8.53 (d, ^3^*J*_HH_ = 4.9 Hz, 1 H, H-6). –^13^C{^1^H}-NMR (CDCl_3_): δ = 21.5 (CH_3_), 116.5 (C-3), 123.9 (*o*-Ph), 126.5 (C-5), 129.5 (*m*-Ph), 132.4 (*p*-Ph), 149.6 (C-6), 150.6 (C-4), 152.9 (*i*-Ph), 163.4 (C-2). Chromatography was carried out using silica as stationary phase.

2-*t*-butylazo-pyridine (**1c**): Method A: A round-bottom Schlenk flask was charged with 2-aminopyridine (4.50 g, 47.8 mmol), lithium (331 mg, 47.8 mmol), excess *t*-BuNO (12.5 g, 143 mmol), and Et_2_O (50 mL). The mixture was stirred for 3 d in the dark. Evaporation of volatiles and purification of the resulting crude product by flash chromatography yielded **1c** (4.24 g, 26.0 mmol, 54%) as a yellow oil. Method B: A solution of 2-nitrosopyridine (1.00 g, 9.25 mmol) and *t*-butylamine (1.09 mL, 765 mg, 10.5 mmol) in degassed methylene chloride (40 mL) was stirred for 24 h in the dark. Evaporation of volatiles from the resulting yellow solution and flash chromatography (eluent petroleum ether/ethyl acetate 9:1) of the residue afforded **1c** as a yellow oil (1.28 g, 7.84 mmol, 85%). The products obtained by either method were characterized by ^1^H and ^13^C NMR spectroscopy and used as is for further reactions. –^1^H NMR (CDCl_3_): δ = 1.33 (s, 9 H, *t*-Bu), 7.29 (ddd, ^3^*J*_HH_ = 7.4 Hz, 4.8 Hz, ^4^*J*_HH_ = 1.1 Hz, 1 H, H-5), 7.43 (ddd, ^3^*J*_HH_ = 8.0 Hz, ^4^*J*_HH_ = 1.1 Hz, 1 H, H-3), 7.77 (ddd, ^3^*J*_HH_ = 8.0 Hz, 7.4 Hz, ^4^*J*_HH_ = 1.9 Hz, 1 H, H-4), 8.59 (ddd, ^3^*J*_HH_ = 4.8 Hz, ^4^*J*_HH_ = 1.9 Hz, ^5^*J*_HH_ = 1.1 Hz, 1 H, H-6). –^13^C{^1^H} NMR (CDCl_3_): δ = 26.9 (CH_3_), 69.0 (NC), 114.2 (3-C), 124.7 (5-C), 138.4 (4-C), 149.2 (6-C), 162.8 (2-C).

2-(*t*-butylazo)-4-methylpyridine (**1d**): A suspension of LDA (1.87 g, 17.5 mmol) in Et_2_O (30 mL) was added slowly to a cooled (0 °C) solution of 4-methyl-2-aminopyridine (1.72 g, 15.9 mmol) in Et_2_O (20 mL). The mixture was warmed to rt, and *t-*BuNO (3.44 mL, 3.16 g, 36.3 mmol) was added. After 3 d, the reaction mixture was quenched with water (50 mL). The organic layer was separated, washed with water, and dried with brine and then MgSO_4_. Evaporation of volatiles and purification of the residue by column chromatography (eluent PE/ethyl acetate 1:1) afforded 1d as a yellow oil (422 mg, 2.38 mmol, 15%) that was used as is for further reactions. –^1^H NMR (CDCl_3_): δ = 1.33 (s, 9 H, *t*-Bu), 2.36 (s, 3 H, CH_3_), 7.10 (dq, ^3^*J*_HH_ = 5.0 Hz, ^4^*J*_HH_ = 0.8 Hz, 1 H, H-5), 7.23 (m, 1 H, H-3), 8.43 (d, ^3^*J*_HH_ = 5.0 Hz, 1 H, H-6). –^13^C{^1^H} NMR (CDCl_3_): δ = 20.1 (CH_3_), 25.8 (C*C*H_3_), 67.8 (*C*CH_3_), 113.6 (3-C), 124.6 (5-C), 147.8 (4-C), 148.8 (6-C), 161.9 (2-C).

4-(*t*-butyl)-2-(*t*-butylazo)pyridine (**1e**): A round-bottom Schlenk flask was charged with 4-*t*-butyl-aminopyridine (3.50 g, 23.3 mmol), Na (535 mg, 23.3 mmol), *t-*BuNO (5.46 g, 62.7 mmol) and Et_2_O (ca. 80 mL). The mixture was stirred for 5 d. Water (50 mL) was slowly added. The organic layer of the resulting biphasic system was separated, washed with water, and dried with brine and then MgSO_4_. Evaporation of volatiles and purification of the residue by column chromatography (eluent PE/ethyl acetate 5:1) afforded **1e** as a yellow oil (4.22 g, 19.2 mmol, 82%) that was used as is. –^1^H NMR (CDCl_3_): δ = 1.34 (s, 9 H, *t*-Bu), 1.39 (s, 9 H, *t*-Bu), 7.31 (dd, ^3^*J*_HH_ = 5.3 Hz, ^4^*J*_HH_ = 1.8 Hz, 1 H, H-5), 7.47 (d, ^4^*J*_HH_ = 1.8 Hz, 1 H, H-3), 8.52 (d, ^3^*J*_HH_ = 5.3 Hz, 1 H, H-6). –^13^C{^1^H} NMR (CDCl_3_): δ = 27.3 (C*C*H_3_), 30.9 (C*C*H_3_), 35.5 (*C*CH_3_), 69.2 (N*C*CH_3_), 112.3 (3-C), 122.2 (5-C), 149.4 (6-C), 163.3 (2-C or 4-C), 163.5 (2-C or 4-C).

2-(2,6-diisopropylphenyl)azo-pyridine (**1f**): Nitrosopyridine (2.50 g, 23.1 mmol) and diisopropylaniline (4.10 g, 23.1 mmol) were dissolved in methylene chloride. A few drops of degassed acetic acid were added, and the reaction mixture was stirred in the dark for 3 d. Solvent and catalyst were removed under reduced pressure and the residue was purified by column chromatography to give a red oil (yield 1.11 g, 4.16 mmol, 18%) that was characterized by ^1^H NMR spectroscopy and used as is for further syntheses. The ^1^H NMR spectrum displayed additional signals, which were assigned to a second stereoisomer (presumably rotamer or Z-isomer, approx. 17%). –^1^H NMR (CDCl_3_): δ = 1.12 (d, ^3^*J*_HH_ = 6.9 Hz, 12 H, CH_3_), 3.09 (*sept*, ^3^*J*_HH_ = 6.9 Hz, 2 H, CH), 7.16 –7.28 (*m*, 3 H, CH_Dipp_), 7.38 (ddd, ^3^*J*_HH_ = 7.5 Hz, 4.8 Hz, ^4^*J*_HH_ = 1.2 Hz, 1 H, H-5), 7.68 (d, ^3^*J*_HH_ = 7.6 Hz, 1 H, H-3), 7.87 (ddd, ^3^*J*_HH_ = 7.5 Hz, 7.6 Hz, ^4^*J*_HH_ = 2.0 Hz, 1 H, H-4), 8.70 (ddd, ^3^*J*_HH_ = 4.8 Hz, ^4^*J*_HH_ = 2.0 Hz, ^5^*J*_HH_ = 0.8 Hz, 1 H, H-6).

3-bromo-2-(pyridin-2-yl)-2,3-dihydro-[1,2,4,3]triazaphospholo[4,5-a]pyridine (**2a**): A round-bottom Schlenk flask was charged with azopyridine 1a (500 mg, 2.71 mmol) and methylene chloride (30 mL). Cyclohexene (668 mg, 8.13 mmol) and PBr_3_ (807 mg, 2.98 mmol) were subsequently added slowly, and the resulting mixture was stirred for 12 h. Volatiles were removed under reduced pressure. The residue was washed with pentane (20 mL) to furnish 2a (714 mg, 2.42 mmol, 89%) as a yellowish powder whose low solubility in organic solvents precluded its characterization by ^13^C NMR spectroscopy. –^1^H NMR (CDCl_3_): δ = 6.43 (t, ^3^*J*_HH_ = 5.9 Hz, 1 H), 6.94–7.16 (m, 3 H), 7.56 –7.83 (m, 3 H), 8.41 (d, ^3^*J*_HH_ = 4.6 Hz, 1 H). –^31^P{^1^H} NMR (CDCl_3_): δ = 135.0 (s). C_10_H_8_BrN_4_P (295.08 g/mol), calcd. C 40.70 H 2.73 N 18.99, found C 39.93 H 2.73 N 18.53.

3-bromo-7-methyl-2-phenyl-2,3-dihydro-[1,2,4,3]triazaphospholo[4,5-a]pyridine (**2b**): The reaction of azopyridine 1b (893 mg, 4.53 mmol), cyclohexene (1.12 g, 13.6 mmol) and PBr_3_ (1.35 g, 4.98 mmol) was carried out as described for 2a to yield 2b (1.27 g, 4.12 mmol, 91%) as a yellowish solid. –^1^H NMR (CDCl_3_): δ = 2.28 (s, 3 H, CH_3_), 6.36 (dd, ^3^*J*_HH_ = 7.2 Hz, ^4^*J*_HH_ = 1.4 Hz, 1 H, H-6), 6.95 (m, 1 H, H-8), 7.26 (m, 1 H, *p*-Ph), 7.44 (m, 2 H, *o*-Ph), 7.64–7.69 (m, 3 H, *m*-Ph and H-5). –^13^C{^1^H} NMR (CDCl_3_): δ = 22.1 (d, ^5^*J*_PC_ = 0.9 Hz, CH_3_), 113.4 (d, ^3^*J*_PC_ = 0.7 Hz, 8-C), 115.5 (d, ^3^*J*_PC_ = 12.6 Hz, 6-C), 120.2 (d, ^3^*J*_PC_ = 14.8 Hz, *o*-Ph), 126.4 (d, ^5^*J*_PC_ = 3.1 Hz, *p*-Ph), 128.4 (d, ^2^*J*_PC_ = 14.8 Hz, 5-C), 130.0 (d, ^3^*J*_PC_ = 0.6 Hz, *m*-Ph), 141.4 (d, ^2^*J*_PC_ = 9.0 Hz, *i*-Ph), 143.1 (d, ^4^*J*_PC_ = 4.3 Hz, 7-C), 150.0 (d, ^2^*J*_PC_ = 11.5 Hz, 9-C). –^31^P{^1^H} NMR (CDCl_3_): δ = 138.6 (s). –C_12_H_11_BrN_3_P (308.12 g/mol): calcd. C 46.78 H 3.60 N 13.64, found C 47.10 H 3.66 N 14.45.

3-bromo-2-(*t*-butyl)-2,3-dihydro-[1,2,4,3]triazaphospholo[4,5-a]pyridine (**2c**): The reaction of azopyridine 1c (240 mg, 1.47 mmol), cyclohexene (362 mg, 4.41 mmol, 3 equivs.) and PBr_3_ (439 mg, 1.62 mmol) in methylene chloride (20 mL) were carried out as described for 2a yielding 2c (344 mg, 1.26 mmol, 86%) as a yellow solid. –^1^H NMR (CDCl_3_): δ = 1.68 (d, ^4^*J*_PH_ = 1.9 Hz, 9 H, *t*-Bu), 6.58 (td, ^3^*J* = 6.7 Hz, ^4^*J* = 0.9 Hz, 1 H, H-2), 7.10 (m, 1 H, H-3), 7.24 (m, 1 H, H-4), 7.89 (m, 1 H, H-1). –^13^C{^1^H} NMR (CDCl_3_): δ = 29.1 (d, ^3^*J*_PC_ = 12.0 Hz, C*C*H_3_), 60.8 (d, ^2^*J*_PC_ = 5.1 Hz, N*C*CH_3_), 112.4 (d, ^2^*J*_PC_ = 12.1 Hz, 2-C), 116.4 (s, 4-C), 129.4 (d, ^2^*J*_PC_ = 14.0 Hz, 1-C), 131.1 (d, ^4^*J*_PC_ = 3.5 Hz, 3-C), 149.5 (d, ^2^*J*_PC_ = 9.8 Hz, 5-C). –^31^P{^1^H} NMR (CDCl_3_): δ = 154.9 (s). C_9_H_13_BrN_3_P (274.10 g/mol): calcd. C 39.44 H 4.78 N 15.33, found C 38.49 H 4.81 N 14.70.

3-bromo-2-(*t*-butyl)-7-methyl-2,3-dihydro-[1,2,4,3]triazaphospholo[4,5-a]pyridine (**2d**): The reaction of azopyridine 1d (240 mg, 1.35 mmol), cyclohexene (343 mg, 4.06 mmol) and PBr_3_ (402 mg, 1.49 mmol) in methylene chloride (20 mL) and the purification of the crude product was carried out as described for 2a yielding 2d (319 mg, 1.11 mmol, 82%) as a yellow solid. –^1^H NMR (CDCl_3_): δ = 1.67 (d, ^4^*J*_PH_ = 1.8 Hz, 9 H, *t*-Bu), 2.27 (s, 3 H, CH_3_), 6.45 (dd, ^3^*J*_HH_ = 7.2 Hz, ^4^*J*_PH_ = 1.3 Hz, 1 H, H-6), 7.05 (s, 1 H, H-8), 7.81 (dd, ^3^*J*_HH_ = 7.2 Hz, ^3^*J*_PH_ = 4.1 Hz, 1 H, H-5). –13C{^1^H} NMR (CDCl_3_): δ = 22.0 (CH_3_), 29.4 (d, ^3^*J*_PC_ = 12.0 Hz, C*C*H_3_), 61.1 (d, ^2^*J*_PC_ = 5.0 Hz, N*C*CH_3_), 113.9 (s, 8-C), 116.2 (d, ^3^*J*_PC_ = 11.9 Hz, 6-C), 129.0 (d, ^2^*J*_PC_ = 14.1 Hz, 5-C), 143.3 (br, 7-C), 150.5 (^2^*J*_PC_ = 10.0 Hz, 9-C). –^31^P{^1^H} NMR (CDCl_3_): δ = 154.6 (s). –C_10_H_15_BrN_3_P (288.13 g/mol): calcd. C 41.69 H 5.25 N 14.58, found C 38.86 H 4.94, N 13.07.

3-Bromo-2,7-di-*t*-butyl-2,3-dihydro-[1,2,4,3]triazaphospholo[4,5-a]pyridine (**2e**): The reaction of 1e (3.50 g, 16.0 mmol), cyclohexene (3.94 g, 48.0 mmol) and PBr_3_ (4.76 g, 17.6 mmol) in methylene chloride (100 mL) and the purification of the crude product was carried out as described for 2a yielding 2e (4.14 g, 12.5 mmol, 78%) as a yellow solid. –^1^H NMR (CDCl_3_): δ = 1.26 (s, 9 H, 7-*t*-Bu), 1.67 (d, ^4^*J*_PH_ = 1.3 Hz, 9 H, 2-*t*-Bu), 6.65 (dd, ^3^*J*_HH_ = 7.5 Hz, ^4^*J*_PH_ = 1.7 Hz, 1 H, H-6), 7.13 (br s, 1 H, H-8), 7.86 (ddd, ^3^*J*_HH_ = 7.5 Hz, ^3^*J*_PH_ = 4.0 Hz, ^5^*J*_HH_ = 0.7 Hz, 1 H, H-5). –13C{^1^H} NMR (CDCl_3_): δ = 29.1 (d, ^3^*J*_PC_ = 12.1 Hz, NC*C*H_3_), 29.5 (s, C*C*H_3_), 35.2 (s, *C*CH_3_), 60.8 (d, ^2^*J*_PC_ = 5.0 Hz, N*C*CH_3_), 110.0 (s, 6-C), 112.6 (d, ^3^*J*_PC_ = 11.8 Hz, 8-C), 128.6 (d, ^2^*J*_PC_ = 13.5 Hz, 5-C), 150.6 (d, ^2^*J*_PC_ = 10.0 Hz, 9-C), 155.0 (d, ^4^*J*_PC_ = 3.5 Hz, 7-C). –^31^P{^1^H} NMR (CDCl_3_): δ = 156.4. –C_13_H_21_BrN_3_P (330.21 g/mol): calcd. C 47.29 H 6.41 N 12.73, found C 46.88 H 6.46 N 12.66.

3-Bromo-2-(2,6-diisopropylphenyl)-2,3-dihydro-[1,2,4,3]triazaphospholo[4,5-a]py ridine (**2f**): The reaction of 1e (780 mg, 2.92 mmol), cyclohexene (720 mg, 8.76 mmol,) and PBr_3_ (869 mg, 3.21 mmol) in methylene chloride (30 mL) was carried out as described for 2a yielding 2f (530 mg, 1.40 mmol, 48%) as a brownish solid. –^1^H NMR (CDCl_3_): δ = 1.18 (d, ^3^*J*_HH_ = 6.8 Hz, 6 H, CH_3_), 1.26 (d, ^3^*J*_HH_ = 6.8 Hz, 6 H, CH_3_), 2.94 (sept, ^3^*J*_HH_ = 6.8 Hz, 2 H, CH), 6.63 (dddd, ^3^*J*_HH_ = 7.2, 6.4 Hz, ^4^*J*_HH_ = 1.2 Hz, ^4^*J*_PH_ = 0.6 Hz, 1 H, H-6), 7.15 (dddd, ^3^*J*_HH_ = 9.5, 6.4 Hz, ^4^*J*_HH_ = 1.3 Hz, ^5^*J*_PH_ = 1.3 Hz, 1 H, H-7), 7.24 (dddd, ^3^*J*_HH_ = 9.5 Hz, ^4^*J*_HH_ = 1.3 Hz, ^5^*J*_HH_ = 1.2 Hz, ^6^*J*_PH_ = 2.8 Hz, 1 H, H-8), 7.28 (dd, ^3^*J*_HH_ = 7.7 Hz, ^5^*J*_PH_ = 0.5 Hz, 2 H, CH), 7.94 (dddd, ^3^*J*_HH_ = 7.2 Hz, ^4^*J*_HH_ = 1.3 Hz, ^5^*J*_HH_ = 1.3 Hz, ^3^*J*_PH_ = 4.7 Hz, 1H, H-5). –13C{^1^H} NMR (CDCl_3_): δ = 24.5 (d, ^5^*J*_PC_ = 1.9 Hz, CH_3_), 24.8 (s, CH_3_), 28.7 (CH), 112.0 (d, ^3^*J*_PC_ = 12.1 Hz, 7-C), 116.0 (8-C), 124.3 (d, ^4^*J*_PC_ = 1.2 Hz, *m*-Ph), 129.8 (d, ^2^*J*_PC_ = 12.1 Hz, 5-C), 130.2 (d, ^5^*J*_PC_ = 1.9 Hz, *p*-Ph), 131.4 (d, ^4^*J*_PC_ = 3.8 Hz, 7-C), 133.3 (d, ^3^*J*_PC_ = 7.1 Hz, *i*-Ph), 147.5 (d, ^2^*J*_PC_ = 6.4 Hz, *o*-Ph), 149.0 (d, ^2^*J*_PC_ = 12.1 Hz, 9-C). –^31^P{^1^H} NMR (CDCl_3_): δ = 150.5. –C_17_H_21_BrN_3_P (378.25 g/mol): calcd. C 53.98 H 5.60 N 11.11, found C 53.27 H 5.81 N 10.80.

3-Iodo-2-(pyridin-2-yl)-2,3-dihydro-[1,2,4,3]triazaphospholo[4,5-a]pyridine (**3a**): A solution of PI_3_ (658 mg, 1.60 mmol) in methylene chloride (17 mL) was added dropwise to a solution of 1a (300 mg, 1.63 mmol) in methylene chloride (10 mL). The mixture was stirred for 12 h. Volatiles were removed under reduced pressure and the red residue was washed with pentane. Recrystallization from pyridine furnished 3a as red crystals (224 mg, 656 μmol, 41%). –^1^H NMR (THF-d_8_): δ = 6.68 (t, ^3^*J*_HH_ = 6.5 Hz, 1 H, H-7), 7.11–7.23 (m, 2 H, H-8 and *m*-H), 7.73 (d, ^3^*J*_HH_ = 8.0 Hz, 1 H, *m*-H), 7.86 (t, ^3^*J*_HH_ = 8.0 Hz, 1 H, *p*-H), 7.95–8.04 (m, 1 H, H-6), 8.44 (d, ^3^*J*_HH_ = 4.8 Hz, 1 H, *o*-H), 8.92 (d, ^3^*J*_HH_ = 5.3 Hz, 1 H, H-5). –^31^P{^1^H} NMR (THF-d_8_): δ = 161.6 (s). –C_10_H_8_IN_4_P (342.08 g/mol): calcd. C 35.11 H 2.39 N 16.38, found C 35.26 H 2.57 N 15.99.

2-(*t*-butyl)-3-methyl-2,3-dihydro-[1,2,4,3]triazaphospholo[4,5-a]pyridine (**4**): A 1.6 M solution of MeLi in Et_2_O (1.37 mL, 2.19 mmol) was slowly added at -78 °C to a solution of 2c (600 mg, 2.19 mmol) in THF (25 mL). After the addition was complete, the solution was allowed to ambient temperature. Volatiles were removed under reduced pressure and the residue was distilled under vacuum (bp. 54 °C, 10^−3^ mbar) to afford a dark yellow, highly air-sensitive oil that eventually solidified upon standing (yield 250 mg, 1.19 mmol, 54%). –^1^H NMR (CDCl_3_): δ = 0.88 (d, ^2^*J*_PH_ = 7.2 Hz, 3 H, CH_3_), 1.30 (d, ^4^*J*_PH_ = 1.0 Hz, 9 H, *t*-Bu), 5.66 (dddd, ^3^*J*_HH_ = 7.2, 5.7 Hz, ^4^*J*_HH_ = 0.4 Hz, ^4^*J*_PH_ = 2.1 Hz, 1 H, H-6), 6.50 (dddd, ^3^*J*_HH_ = 9.8, ^4^*J*_HH_ = 1.3 Hz, ^5^*J*_HH_ = 0.4 Hz, ^6^*J*_PH_ = 1.0 Hz, 1 H, H-7), 6.54 (dddd, ^3^*J*_HH_ = 9.8, 5.7 Hz, ^4^*J*_HH_ = 1.3 Hz, ^5^*J*_PH_ = 0.3 Hz, 1 H, H-8), 6.98 (dddd, ^3^*J*_HH_ = 7.2 Hz, ^4^*J*_HH_ = 2.1 Hz, ^5^*J*_HH_ = 1.3 Hz, ^3^*J*_PH_ = 4.6 Hz, 1H, H-5). –13C{^1^H} NMR (CDCl_3_): δ = 20.7 (d, ^1^*J*_PC_ = 37.7 Hz, CH_3_), 29.6 (d, ^3^*J*_PC_ = 9.8 Hz, C*C*H_3_), 56.0 (s, N*C*CH_3_), 105.4 (d, ^3^*J*_PC_ = 6.5 Hz. 6-C), 114.3 (s, 8-C), 131.0 (d, ^4^*J*_PC_ = 1.5 Hz, 7-C), 131.3 (s, 5-C), 149.0 (s, 9-C). –^31^P{^1^H} NMR (CDCl_3_): δ = 88.4 (s). –C_10_H_16_PN_3_ (209.23 g/mol): calcd. C 57.40 H 7.71 N 20.08, found C 58.12 H 8.22 N 20.22.

Bis-{2-(*t*-butyl)-3-methyl-2,3-dihydro-[1,2,4,3]triazaphospholo[4,5-a]pyridine}-{1,5-cyclooctadiene}-nickel(0) (**5**): Addition of a solution of **4** (76 mg, 0.363 mmol) in THF (10 mL) to a solution of Ni(COD)_2_ (50 mg, 0.182 mmol) in THF (7 mL) immediately produced a deep red solution. After stirring for an additional 2 h, the solvent was removed under reduced pressure, the residue suspended in hexane, and filtrated over celite. Storage of the filtrate at −28 °C for 1 week produced a crystalline material identified as a diastereomeric mixture of *rac*- and *meso*-**5** (yield 62 mg, 0.106 mmol, 58%). Manual selection furnished a crystal of *rac*-**5** suitable for a single-crystal XRD study. –^1^H NMR (C_6_D_6_): major diasteromer (80%): δ = 1.30 (s, 6 H_,_ CH_3_), 1.49 (s, 18 H, *t*-Bu), 1.95 (m, 2 H, CH_2_), 2.13 (m, 2 H, CH_2_), 2.29 (m, 2 H, CH_2_), 2.43 (m, 2 H, CH_2_), 4.50 (m, 4 H, =CH), 5.22 (t, ^3^*J*_HH_ = 6.4 Hz, 2 H, H-7), 5.92 (ddd, 2 H, ^3^*J*_HH_ = 9.7, 6.2 Hz, ^4^J_HH_ = 1.0 Hz, H-6), 6.27 (d, 1 H, ^3^*J*_HH_ = 9.6 Hz, H-8), 6.60 (br m, 1 H, H-5); minor diastereomer: δ = 1.36 (s, 6 H_,_ CH_3_), 1.43 (s, 18 H, *t*-Bu), 2.15 (m, 4 H, CH_2_), 2.29 (m, 4 H, CH_2_), 4.37 (m, 2 H, =CH), 4.67 (m, 2 H, =CH), 5.24 (t, ^3^*J*_HH_ = 6.4 Hz, 2 H, H-7), 5.97 (ddd, 2 H, ^3^*J*_HH_ = 9.7, 6.2 Hz, ^4^J_HH_ = 1.0 Hz, H-6), 6.29 (d, 1 H, ^3^*J*_HH_ = 9.6 Hz, H-8), 6.78 (br m, 1 H, H-5). –13C{^1^H} NMR (C_6_D_6_): major diastereomer: δ = 23.3 (m, CH_3_), 29.3 (m, CH_2_), 31.0 (C*C*H_3_), 32.9 (m, CH_2_), 56.6 (N*C*CH_3_), 88.2 (br, =CH), 89.0 (br, =CH), 105.1 (m, 7-C), 116.4 (8-C), 129.0 (s, 6-C), 130.5 (m, 5-C), 145.2 (9-C); minor diastereomer: δ = 23.9 (m, CH_3_), 30.8 (m, CH_2_), 31.0 (m, C*C*H_3_), 29.3 (m, CH_2_), 31.0 (m, C*C*H_3_), 56.9 (N*C*CH_3_), 87.4 (br, =CH), 87.9 (br, =CH), 104.9 (m, 7-C), 116.39 (8-C), 129.2 (s, 6-C), 131.1 (m, 5-C), 145.4 (9-C). –^31^P{^1^H} NMR (C_6_D_6_): δ = 131.0 (major diastereomer, 80%), 132.3 (minor diastereomer, 20%).

Crystallographic Studies. Single-crystal X-ray diffraction data were measured on a Bruker Kappa APEX2 Duo diffractometer at 135(2) K for 3a and 2f, 140(2) K for 2c-e, and 145(2) K for 4 and 5, respectively, using Cu-Kα radiation (λ = 1.54178 Å) for 5 and Mo-$K_{\alpha }$ radiation (λ = 0.71073 Å) for the remaining compounds. The structures were solved by direct methods (SHELXS-2014 [24]) or dual space/intrinsic methods (SHELXT [25]) and refined with a full-matrix-least-squares scheme on *F*^2^ (SHELXL-2014 [24]). Semi-empirical absorption corrections from equivalents were applied for **2c**–**f** and **4**, and numerical absorption corrections for **3a**, **5**. Nonhydrogen atoms were refined anisotropically. The Br-atom of **2f** was disordered over two positions and was refined using restraints, and **2d** was refined as a 2-component twin, BASF = 0.251 (**5**). Further crystallographic data and details on the structure solution are given in the Supplementary Material. CCDC 2183389 (**3a**), 2183390 (**2f**), 2183391 (**2c**), 2183392 (**2e**), 2183393 (**2d**), 2183394 (**4**), and 2183395 (**5**) contain the crystallographic data for this paper, which can be obtained free of charge from the Cambridge Crystallographic Data Centre via www.ccdc.cam.ac.uk/data_request/cif (accessed on 13 July 2022).

## 4. Conclusions

The cycloaddition of PBr_3_ with azopyridines, which are easily available from the condensation of appropriate primary amine- and nitroso-components, provides a modular synthetic pathway to tailored pyridine-annellated 1,3,4,2-triazaphospholenes. Using PI_3_ as a phosphorus source is also feasible, but in most cases synthetically less convenient due to tedious work-up procedures. Post-functionalization of the P–Br bond in a cycloaddition product is demonstrated by the exemplary synthesis of a P-alkylated triazaphospholene, which is further shown to be capable of acting as a P-donor ligand toward a transition metal center. Even if this ligand was to date only obtained in racemic form, its annellated triazaphospholene framework makes a new structural motif for the design of chiral phosphines. Crystallographic studies indicate that the isoelectronic relation of annellated P-bromo-triazaphospholenes with the better-known diazaphospholenes is reflected in a similar, albeit somewhat less pronounced, ionic polarization of the P–Br bond.

## Figures and Tables

**Table 1:** Selected intramolecular distances (in Å) for **2c**–**f**, **3a**, **4**, **5**.

	X	R2	R5	P–X ^1^	P–N	N–N
**2c**	Br	*t*-Bu	H	2.6837 (6)	1.649 (2) 1.692 (2)	1.385 (2)
**2d**	Br	*t*-Bu	Me	2.8950 (16)	1.665 (5) 1.697 (5)	1.364 (6)
**2e**	Br	*t*-Bu	*t*-Bu	2.5683 (6)	1.659 (2) 1.696 (2)	1.394 (2)
**2f**	Br	Dipp	H	2.4695 (12)/ 2.516 (6) ^2^	1.6611 (13) 1.7031 (14)	1.397 (2)
**3a** ^3^	I	Py	H	2.822 (2) 2.834 (3)	1.672 (10) 1.707 (10) 1.681 (10) 1.694 (9)	1.397 (12) 1.362 (12)
**4**	Me	*t*-Bu	H	1.845 (2)	1.702 (2) 1.739 (2)	1.429 (2)
**5** ^4^	Me	*t*-Bu	H	1.848 (2) 1.839 (2)	1.746 (2) 1.727 (2) 1.739 (2) 1.754 (2)	1.425 (2) 1.428 (2)

^1^ Additional intermolecular distances: P–Br 3.7229 (7) (**2c**), 3.6133 (16) (**2d**), 3.8782 (6) (**2e**), 4.2562 (15) (**2f**). ^2^ Values refer to two disordered sites of the Br atom with occupancies of 0.78 (2):0.22 (2). ^3^ Values for two crystallographically independent molecules. ^4^ Ni–P 2.1440 (**5**), 2.1554 (**6**) Å.

## Data Availability

Data is contained within the article or supplementary material. CCDC 2183389 (**3a**), 2183390 (**2f**), 2183391 (**2c**), 2183392 (**2e**), 2183393 (**2d**), 2183394 (**4**), and 2183395 (**5**) contain the crystallographic data for this paper, which can be obtained free of charge from the Cambridge Crystallographic Data Centre via www.ccdc.cam.ac.uk/data_request/cif (accessed on 13 July 2022).

## Supplementary Materials

The following supporting information can be downloaded at: https://www.mdpi.com/article/10.3390/molecules27154747/s1, Table S1. X-ray Details for **2c**–**f**, **3a**, **4**, **5**; Table S2. Selected endocyclic distances (in Å) for **2c**–**f**, **3a**, **4**, **5**. Figure S1. Representation of the molecular structures of **2c**, **2f**, and **3a**. Figure S2: ^1^H NMR spectra for **2b**–**f**, **3a**, **4**, **5**. Figure S3: ^13^C{^1^H} NMR spectra for **2b**–**f**, **4**, **5**. Figure S4: ^31^P{^1^H} NMR spectra for **2b**–**f**, **3a**, **4**, **5**.

## References

[1] Gudat D. (2010). Recent Developments in the Chemistry of N-Heterocyclic Phosphines. Top. Heterocycl. Chem..

[2] Gudat D. (2010). Diazaphospholenes: *N*-Heterocyclic Phosphines between Molecules and Lewis Pairs. Acc. Chem. Res..

[3] Gudat D. (2016). A very peculiar family of N-heterocyclic phosphines: Unusual structures and the unique reactivity of 1,3,2-diazaphospholenes. Dalton Trans..

[4] Chong C.C., Hirao H., Kinjo R. (2014). A Concerted Transfer Hydrogenolysis: 1,3,2-Diazaphospholene-Catalyzed Hydrogenation of N=N Bond with Ammonia–Borane. Angew. Chem. Int. Ed..

[5] Speed A.W.H. (2020). Applications of diazaphospholene hydrides in chemical catalysis. Chem. Soc. Rev..

[6] Reed J.H., Klett J., Steven C., Cramer N. (2020). Stay positive: Catalysis with 1,3,2-diazaphospholenes. Org. Chem. Front..

[7] Schmidpeter A. (1986). Synthetic routes to five-membered aromatic phosphorus heterocycles. Phosphorus Sulfur Rel. Elem..

[8] Tien C.-H., Adams M.R., Ferguson M.J., Johnson E.R., Speed A.W.H. (2017). Hydroboration Catalyzed by 1,2,4,3-Triazaphospholenes. Org. Lett..

[9] Panova Y.S., Sheyanova A.V., Zolotareva N.V., Sushev V.V., Arapova A.V., Novikov A.S., Baranov E.V., Fukin G.K., Kornev A.N. (2018). 2,2′-Azobispyridine in Phosphorus Coordination Chemistry: A New Approach to 1,2,4,3-Triazaphosphole Derivatives. Eur. J. Inorg. Chem..

[10] Benkő Z., Burck S., Gudat D., Nieger M., Nyulászi L., Shore N. (2008). Pyrido-Annellated Diazaphospholenes and Phospholenium Ions. Dalton Trans..

[11] Reeske G., Cowley A.H. (2007). One-Step Redox Route to N-Heterocyclic Phosphenium Ions. Inorg. Chem..

[12] Dube J.W., Farrar G.J., Norton E.L., Szekely K.L.S., Cooper B.F.T., Macdonald C.L.B. (2009). A Convenient Method for the Preparation of N-Heterocyclic Bromophosphines: Excellent Precursors to the Corresponding N-Heterocyclic Phosphenium Salts. Organometallics.

[13] Rivarola E., Silvestri A., Alonzo G., Barbieri R. (1985). Synthesis and Structural Studies by Infrared and Mössbauer Spectroscopy of Adducts of Tin (IV) and Organotin (IV) Derivatives with 2,2′-azopyridine. Inorg. Chim. Acta.

[14] Campbell N., Henderson A.W., Taylor D.J. (1953). Geometrical Isomerism of Azo-compounds. J. Chem. Soc..

[15] Krause R.A., Krause K. (1980). Chemistry of Bipyridyl-like Ligands. Isomeric Complexes of Ruthenium (II) with 2-(Phenylazo) pyridine. Inorg. Chem..

[16] Herrmannsdörfer D., Kaaz M., Puntigam O., Bender J., Nieger M., Gudat D. (2015). The Reaction between Diazadienes and Element Tribromides EBr_3_ (E = P, B) Revisited: Metal-Free Synthesis of Halogenated N-Heterocyclic Phosphanes and Boranes. Eur. J. Inorg. Chem..

[17] Alvarez S. (2013). A cartography of the van der Waals territories. Dalton Trans..

[18] Allen F.H., Kennard O., Watson D.G., Brammer L., Orpen A.G., Taylor R. (1987). Tables of Bond Lengths determined by X-Ray and Neutron Diffraction. Part 1. Bond Lengths in Organic Compounds. J. Chem. Soc. Perkin Trans..

[19] Groom C.R., Bruno I.J., Lightfoot M.P., Ward S.C. (2016). Median values returned by queries in the CSD database for phosphines with N_2_PX (X = Br, I) substitution patterns. The Cambridge Structural Database. Acta Cryst..

[20] Burck S., Gudat D., Nieger M., Benkő Z., Nyulászi L., Szieberth D. (2009). Spontaneous Phosphorus-Halogen Bond Cleavage in N-Heterocyclic Halogenophosphanes Revisited: The Case of P-Br and P-I Bonds. Z. Anorg. Allg. Chem..

[21] Median Value and Standard Deviation Returned by a Query in the CSD Database for the P–Ni Distances in Phosphine Complexes of Nickel. https://www.ccdc.cam.ac.uk.

[22] Armarego W.L.F., Chai C.L.L. (2003). Purification of Laboratory Chemicals.

[23] Harris R.H., Becher E.D., Menezes S.M.C.d., Goodfellow R., Granger P. (2002). NMR nomenclature: Nuclear spin properties and conventions for chemical shifts (IUPAC recommendations 2001). Concepts Magn. Reson..

[24] Sheldrick G.M. (2015). Crystal structure refinement with SHELXL. Acta Cryst..

[25] Sheldrick G.M. (2015). SHELXT—Integrated space-group and crystal-structure determination. Acta Cryst..

