# Supplementary material for: Annellated 1,3,4,2-Triazaphospholenes-Simple Modular Synthesis and a First Exploration of Ligand Properties
Source: Molecules. 2022 Jul 25;27(15):4747. doi: 10.3390/molecules27154747 (PMC9330784; doi:10.3390/molecules27154747)
Supplement: Supplementary file 1 [file molecules-27-04747-s001.zip › molecules-1838163-supplementary.pdf]

Table S1. X-Ray Details for **2c-f**, **3a**, **4**, **5**.

	**2c**	**2d**	**2e**	**2f**
empirical formula	$C_9H_{13}BrN_3P$	$C_{10}H_{15}BrN_3P$	$C_{13}H_{21}BrN_3P$	$C_{17}H_{21}BrN_3P$
formula weight (g·mol^{-1})	274.10	288.13	330.21	378.25
T (K)	140(2)	140(2)	140(2)	135(2)
crystal size (mm)	0.14 × 0.13 × 0.09	0.33 x 0.25 x 0.16	0.24 x 0.17 x 0.13	0.43 × 0.40 × 0.11
Wavelength (Å)	0.71073	0.71073	0.71073	0.71073
space group	$P2_1/c$	$P2_1/c$	$P2_1/c$	$P2_1/c$
a (Å)	13.1299(10)	9.6420(10)	13.5105(8)	9.4382(4)
b (Å)	6.7889(5)	18.2770(17)	10.2558(5)	9.5359(5)
c (Å)	12.7409(9)	6.8279(6)	11.4811(5)	19.6502(9)
a (deg)	90	90	90	90
β (deg)	93.186(3)	95.909(3)	99.263(3)	97.338(3)
g (deg)	90	90	90	90
V (Å^3)	1133.94(14)	1196.9(2)	1570.09(14)	1754.07(14)
Z	4	4	4	4
D_c (mg·m^{-3})	1.606	1.599	1.397	1.432
μ (mm^{-1})	3.73	3.54	2.71	2.44
F (000)	552	584	680	776
Θ range (deg)	1.55 to 28.34	2.12 to 28.42	2.51 to 28.31	2.09 to 30.57
reflections collected	14724	2985	17472	23196
unique reflections	2810	2985	3914	5354
R_{int}	0.033	a)	0.038	0.027
max./min transmission	0.7923 / 0.6576	0.6411 / 0.4161	0.7706 / 0.5883	0.8076 / 0.4615
Absorption correction	multi-scan	multi-scan	multi-scan	multi-scan
Data / restraints / parameters	2810 / 0 / 130	2985 / 0 / 138	3914 / 0 / 163	5354 / 9 / 209
G.o.F. on F^2	1.03	1.14	1.02	1.01
*R*1 [I > 2σ(I)]	0.031	0.060	0.030	0.030
w*R*2 (F^2) (for all data)	0.078	0.144	0.063	0.073
largest diff. peak/hole (e Å^{-3})	0.583 / -0.654	2.127 / -2.017	0.438 / -0.437	0.754 / -0.525
CCDC	2183391	2183393	2183392	2183390

a) hklf5-file used

Table S1 (continued).

	**3a**	**4**	**5**
empirical formula	$C_{10}H_8IN_4P$	$C_{10}H_{16}N_3P$	$C_{28}H_{44}N_6NiP_2$
formula weight (g·mol^{-1})	342.07	209.23	585.34
T (K)	135(2)	145(2)	145(2)
crystal size (mm)	0.16 × 0.08 × 0.07	0.21 × 0.17 × 0.06	0.17 x 0.10 x 0.08
Wavelength (Å)	0.71073	0.71073	1.54178
space group	$P\bar{1}$	$P2_1/c$	$P\bar{1}$
a (Å)	7.7397(7)	10.3058(7)	9.8205(8)
b (Å)	12.3466(13)	9.1239(6)	9.9504(9)
c (Å)	13.0301(12)	11.8933(9)	16.1331(19)
a (deg)	77.051(6)	90	99.304(6)
β (deg)	81.565(5)	94.519(4)	99.305(6)
g (deg)	72.276(5)	90	104.971(4)
V (Å^3)	1151.7(2)	1114.84(14)	1468.3(3)
Z	4	4	2
D_c (mg·m^{-3})	1.973	1.247	1.324
μ (mm^{-1})	2.90	0.12	2.19
F (000)	656	448	624
Θ range (deg)	1.61 to 26.33	1.98 to 26.40	2.84 to 66.50
reflections collected	14838	15457	17532
unique reflections	4613	2285	4970
R_{int}	0.070	0.057	0.030
max./min transmission	0.8924 / 0.6117	0.7422/ 0.7136	0.8791 / 0.7371
Absorption correction	numerical	multi-scan	numerical
Data / restraints / parameters	4613/ 0 / 289	2285/ 0 / 128	4970/ 4 / 354
G.o.F. on F^2	1.09	1.03	1.03
*R*1 [I > 2σ(I)]	0.068	0.040	0.030
w*R*2 (F^2) (for all data)	0.176	0.089	0.080
largest diff. peak/hole (e Å^{-3})	2.688 / -1.378	0.241 / -0.291	0.386 / -0.234
CCDC	2183389	2183394	2183395

Table S2. Selected endocyclic distances (in Å) for **2c-f**, **3a**, **4**, **5**.

**2c**	**2d**	**2e**	**2f**
P1–N3 1.6494(18)	P1–N1 1.665(5)	P1–N1 1.6589(18)	P1–N1 1.6611(13)
P1–N1 1.6919(19)	P1–N3 1.697(5)	P1–N3 1.6960(16)	P1–N2 1.7031(14)
N2–N3 1.385(2)	N1–N2 1.364(6)	N1–N2 1.394(2)	N1–N3 1.3971(17)
N2–C5 1.306(3)	N2–C5 1.316(7)	N2–C5 1.307(2)	N3–C1 1.302(2)
N1–C1 1.388(3)	N3–C10 1.382(7)	N3–C13 1.386(2)	N2–C5 1.389(2)
N1–C5 1.394(3)	N3–C5 1.400(7)	N3–C5 1.387(2)	N2–C1 1.3950(19)
C4–C5 1.424(3)	C5–C6 1.421(8)	C5–C6 1.430(3)	C1–C2 1.430(2)
C3–C4 1.342(3)	C6–C7 1.363(8)	C6–C7 1.353(3)	C2–C3 1.345(2)
C2–C3 1.416(4)	C7–C9 1.430(8)	C7–C12 1.436(3)	C3–C4 1.429(3)
C1–C2 1.348(3)	C9–C10 1.344(8)	C12–C13 1.338(3)	C4–C5 1.344(2)

Table S2 (continued).

3a [a)]		4	5	
P1–N1 1.672(10)	P2–N5 1.681(10)	P1–N1 1.7019(15)	P1–N3 1.7267(15)	P2–N4 1.7388(14)
P1–N3 1.707(10)	P2–N7 1.694(9)	P1–N3 1.7385(16)	P1–N1 1.7459(15)	P2–N5 1.7542(15)
N1–N2 1.397(12)	N5–N6 1.362(12)	N1–N2 1.429(2)	N2–N3 1.425(2)	N4–N6 1.4281(19)
N2–C1 1.279(15)	N6–C11 1.312(14)	N2–C5 1.299(2)	N2–C1 1.295(2)	N6–C11 1.287(2)
N3–C2 1.368(15)	N7–C12 1.398(15)	N3–C9 1.378(2)	N1–C5 1.376(2)	N5–C15 1.376(2)
C1–N3 1.417(14)	C11–N7 1.377(13)	N3–C5 1.401(2)	N1–C1 1.396(2)	N5–C11 1.400(2)
C1–C5 1.437(16)	C11–C15 1.418(15)	C5–C6 1.433(2)	C1–C2 1.436(3)	C11–C12 1.440(2)
C4–C5 1.340(17)	C14–C15 1.348(16)	C6–C7 1.349(3)	C2–C3 1.340(3)	C12–C13 1.343(3)
C3–C4 1.413(16)	C13–C14 1.439(16)	C7–C8 1.428(3)	C3–C4 1.435(3)	C13–C14 1.432(3)
C2–C3 1.346(17)	C12–C13 1.333(16)	C8–C9 1.338(3)	C4–C5 1.344(3)	C14–C15 1.345(3)

a) data refer to two crystallographically independent molecules

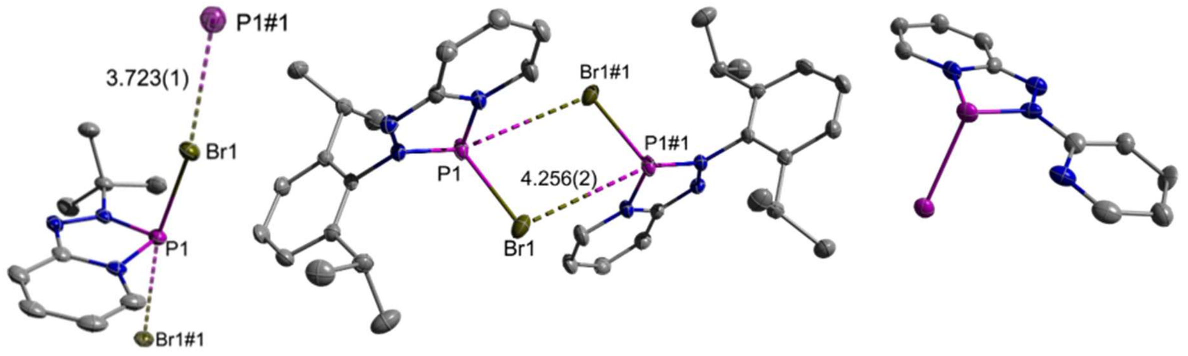

Figure S1. Representation of the molecular structures of **2c** (with contacts to the phosphorus and bromine atoms of adjacent molecules, left), **2f** (dimeric unit, middle), and **3a** (one of two crystallographically independent atoms, right),) in the crystal. Hydrogen atoms were omitted for clarity and intermolecular contacts were drawn as dashed lines with distances in Å. Thermal ellipsoids were drawn at the 50% probability level. Selected distances are listed in Tables 1, S2.

NMR Data

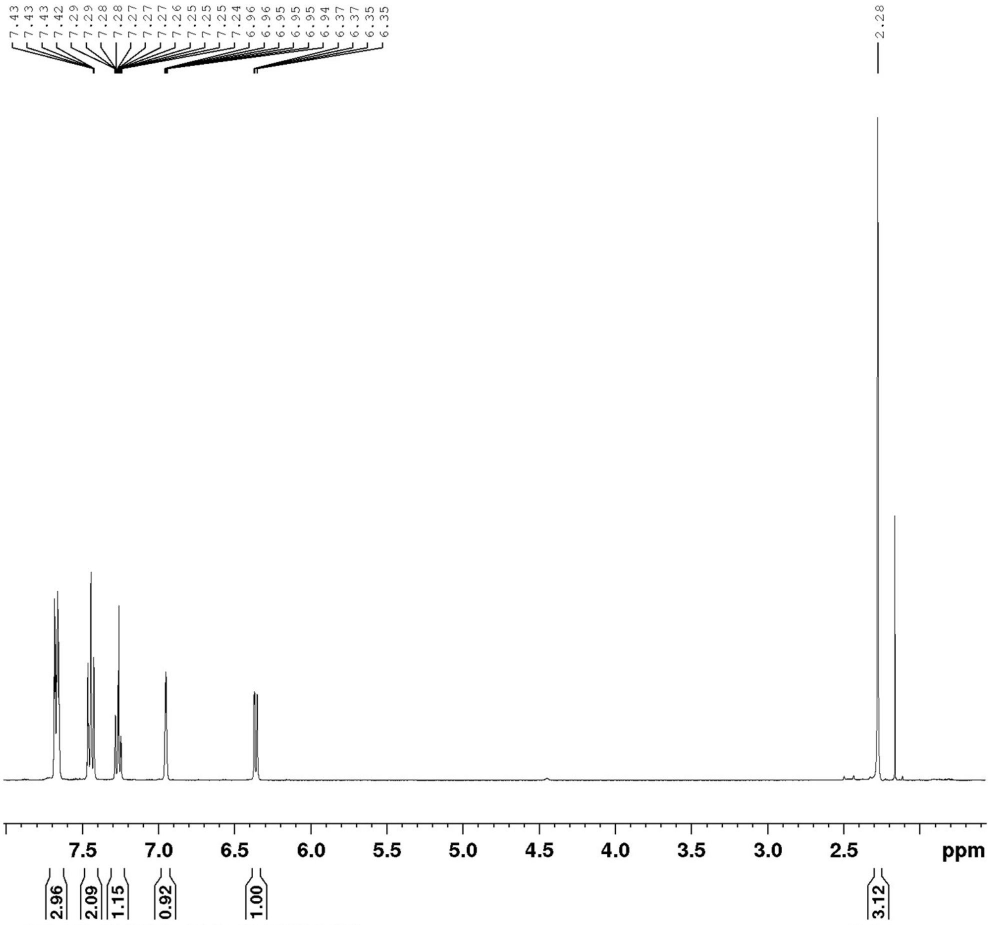

a)

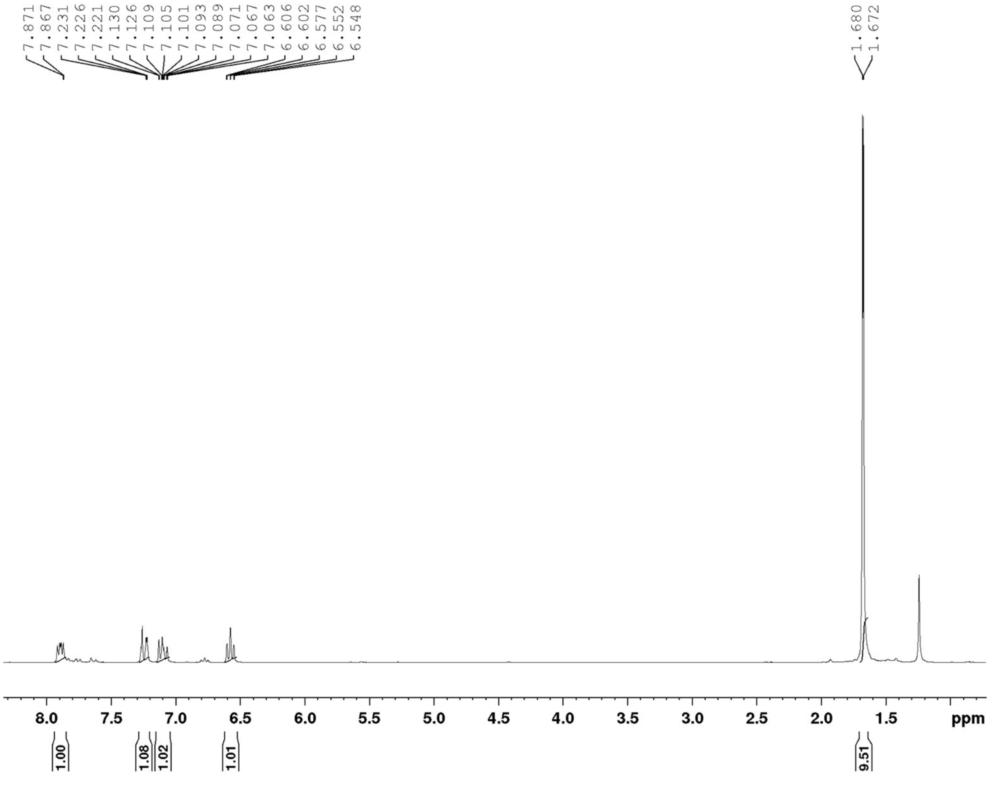

b)

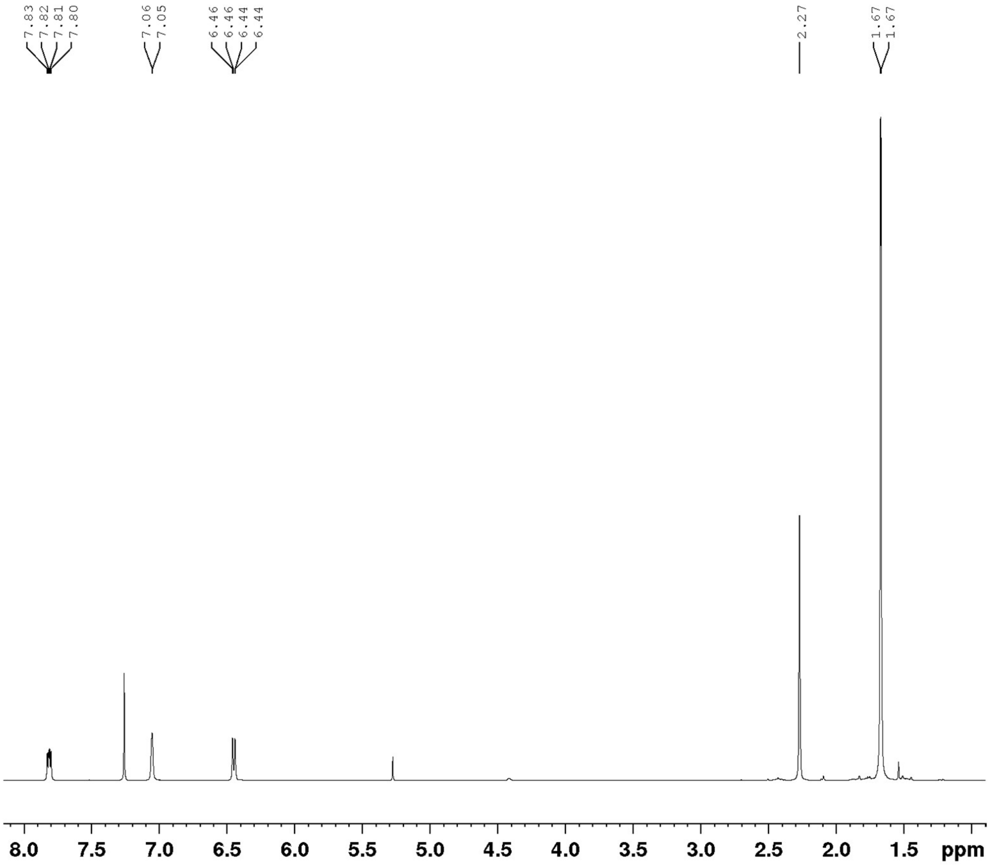
7.83
7.82
7.81
7.80
7.06
7.05
6.46
6.46
6.44
6.44
2.27
1.67
1.67
8.0 7.5 7.0 6.5 6.0 5.5 5.0 4.5 4.0 3.5 3.0 2.5 2.0 1.5 ppm

c)

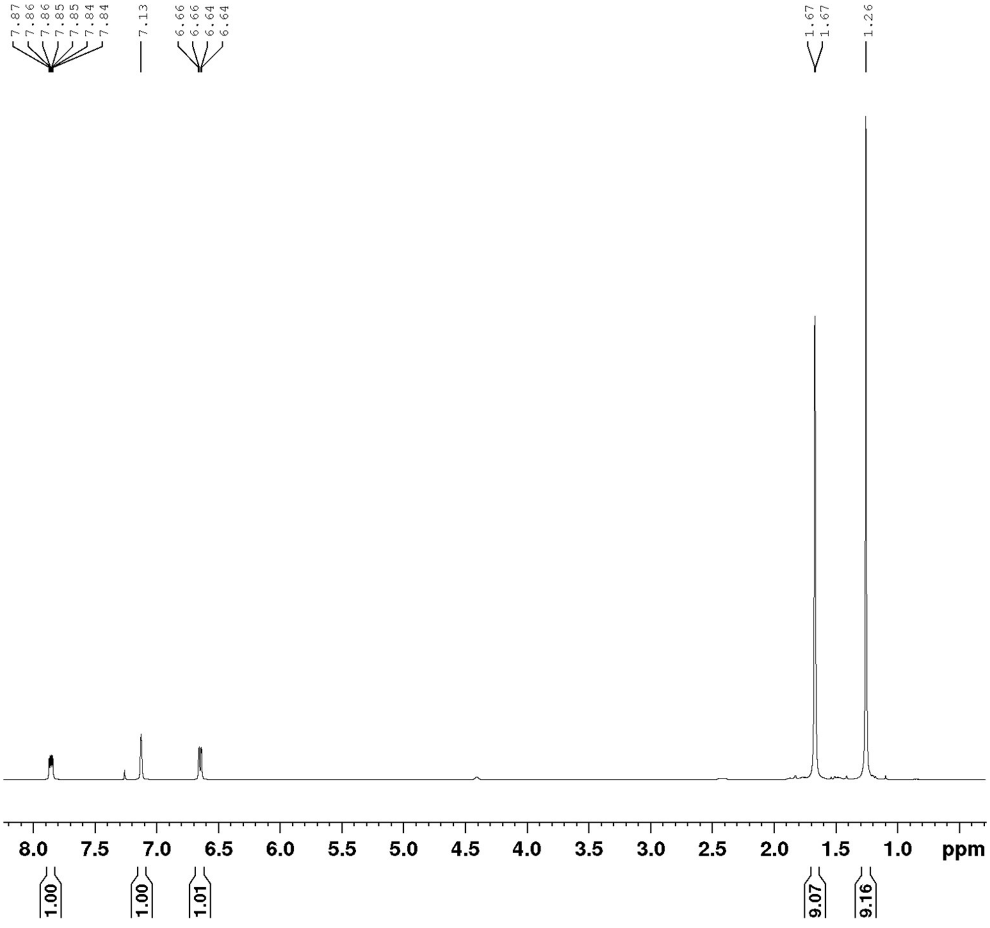
7.87
7.86
7.86
7.85
7.85
7.84
7.84
7.13
6.66
6.66
6.64
6.64
1.67
1.67
1.26
8.0 7.5 7.0 6.5 6.0 5.5 5.0 4.5 4.0 3.5 3.0 2.5 2.0 1.5 1.0 ppm
1.00
1.00
1.01
9.07
9.16

d)

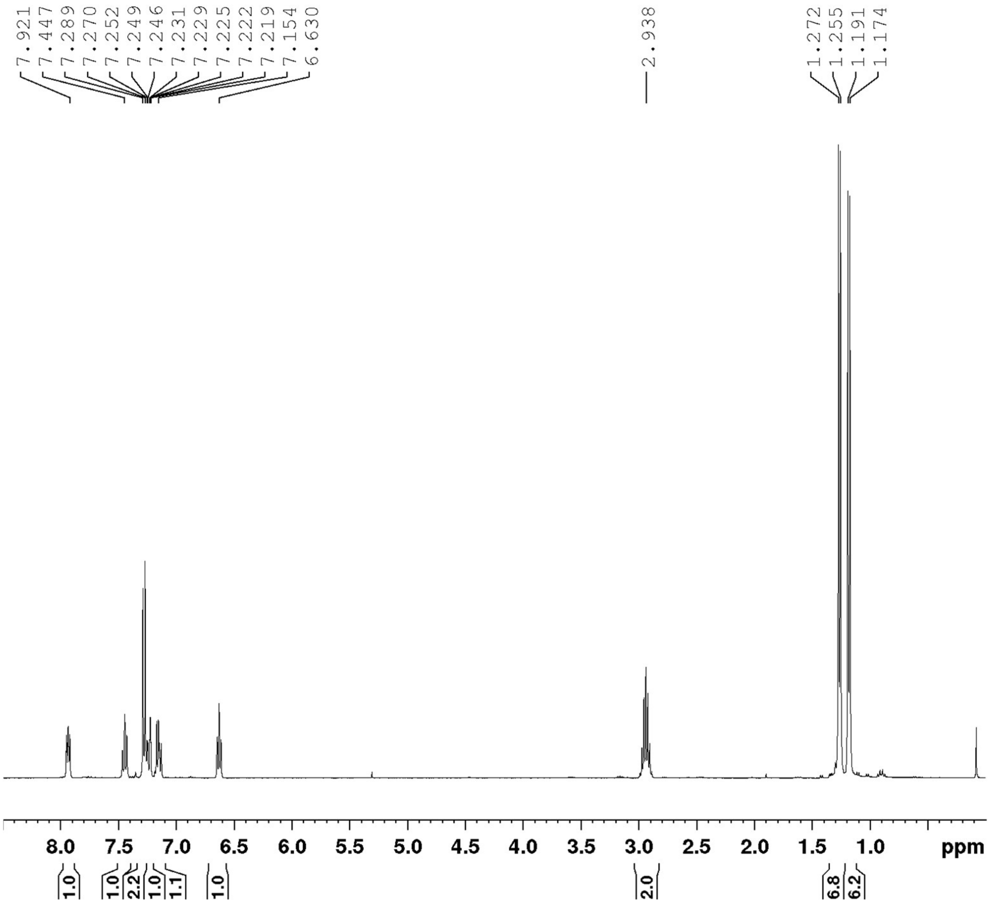
7.921
7.447
7.289
7.270
7.252
7.249
7.246
7.231
7.229
7.225
7.222
7.219
7.154
6.630
2.938
1.272
1.255
1.191
1.174
8.0 7.5 7.0 6.5 6.0 5.5 5.0 4.5 4.0 3.5 3.0 2.5 2.0 1.5 1.0 ppm
1.0
1.0
2.2
1.0
1.1
1.0
2.0
6.8
6.2

e)

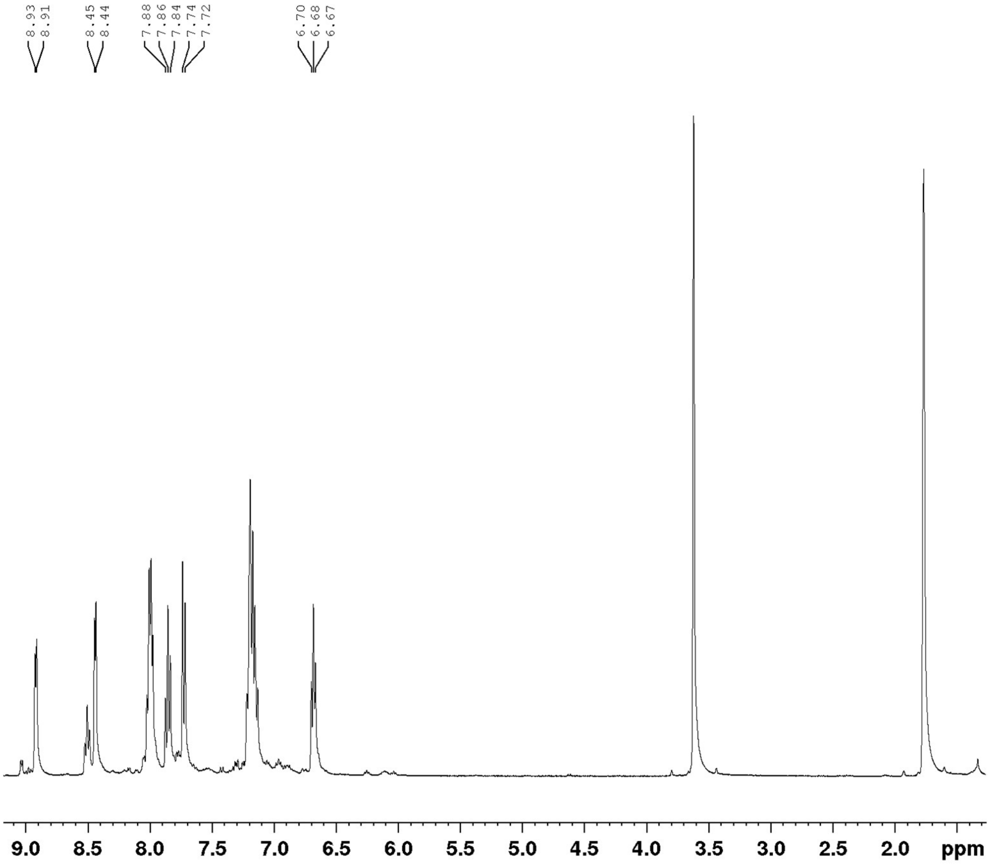
8.93
8.91
8.45
8.44
7.88
7.86
7.84
7.74
7.72
6.70
6.68
6.67
9.0 8.5 8.0 7.5 7.0 6.5 6.0 5.5 5.0 4.5 4.0 3.5 3.0 2.5 2.0 ppm

f)

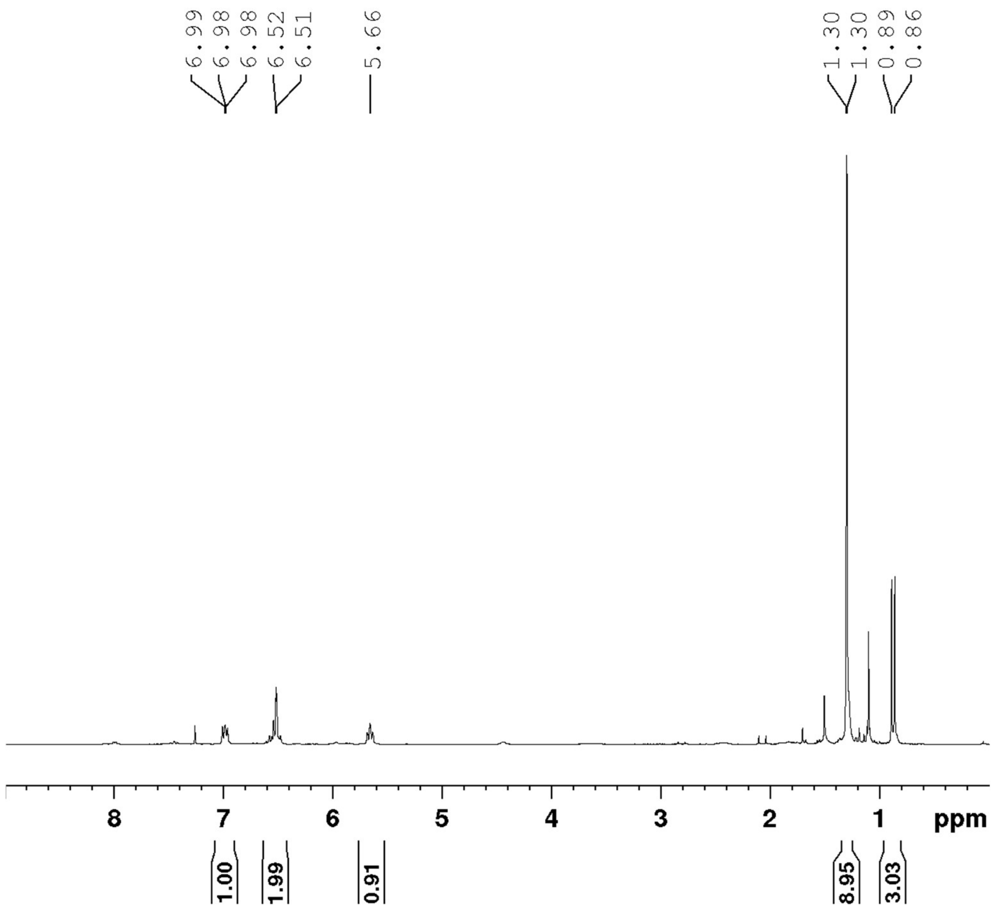

g)

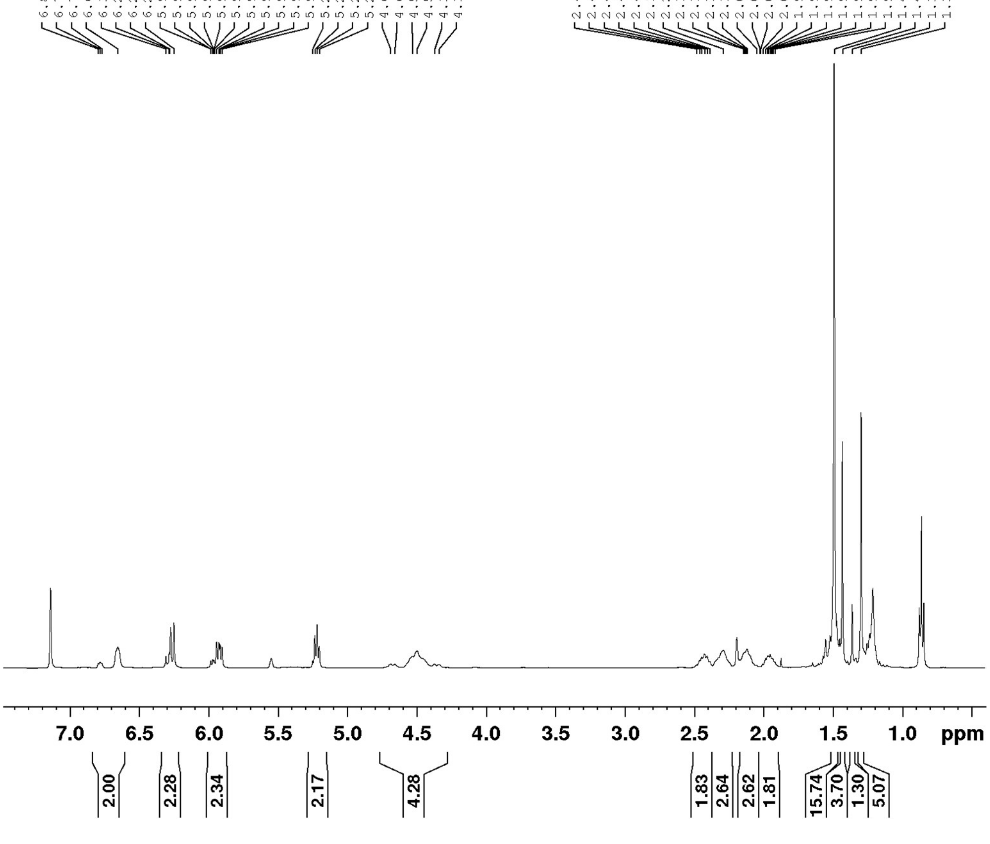

h)

Figure S2: ^1H NMR spectra (in $CDCl_3$) for **2b** (a), **2c** (b), **2d** (c), **2e** (d), **2f** (e), **3a** (f, in THF-d_8), **4** (g), and **5** (h).

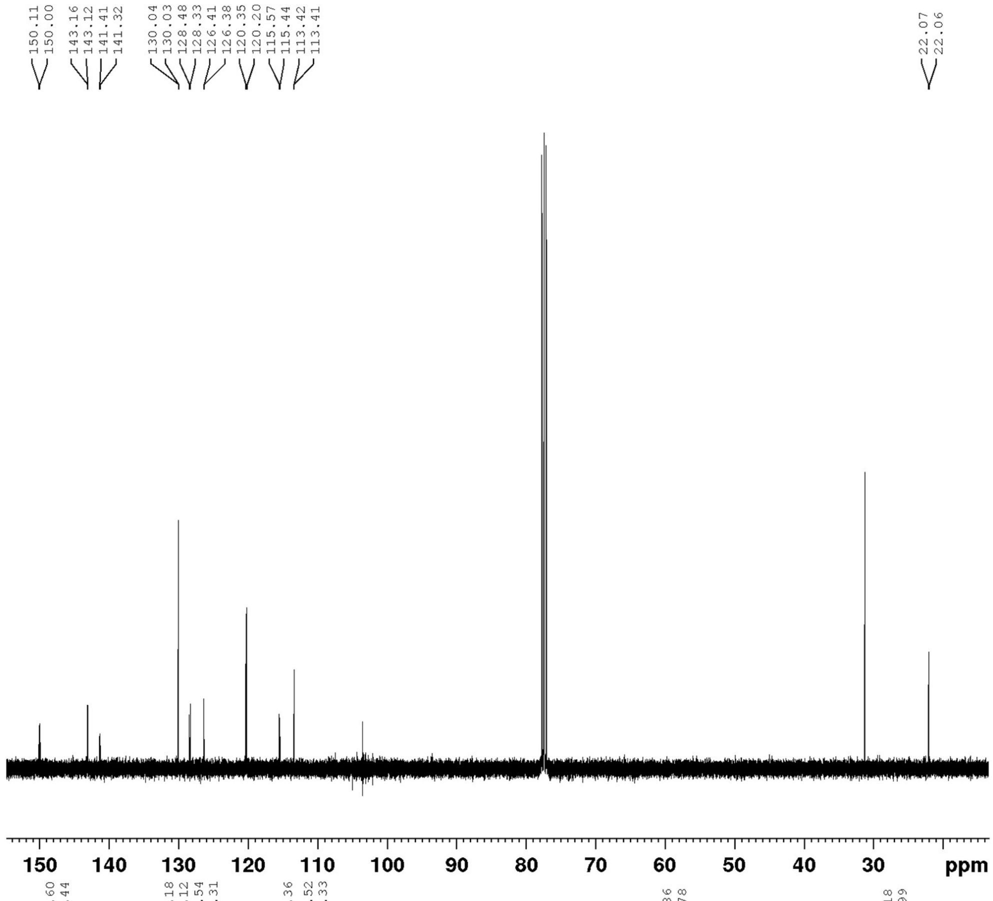
150.11
150.00
143.16
143.12
141.41
141.32
130.04
130.03
128.48
128.33
126.41
126.38
120.35
120.20
115.57
115.44
113.42
113.41
22.07
22.06
150 140 130 120 110 100 90 80 70 60 50 40 30 ppm

a)

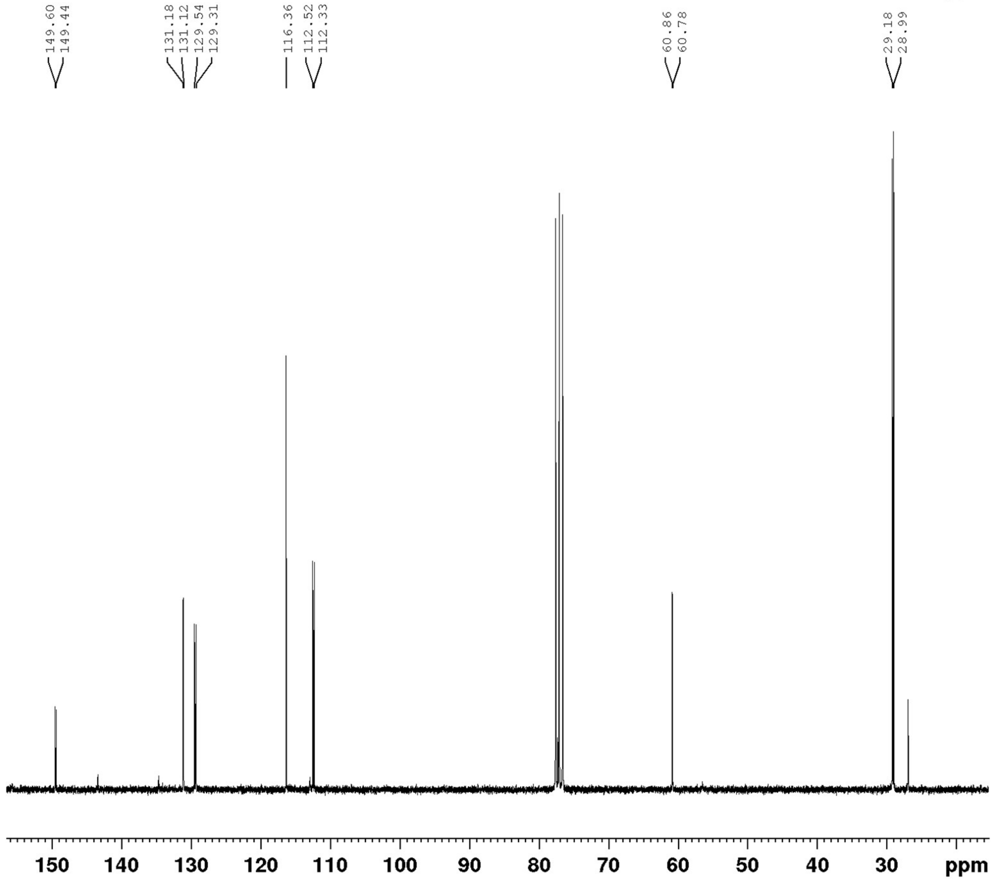
149.60
149.44
131.18
131.12
129.54
129.31
116.36
112.52
112.33
60.86
60.78
29.18
28.99
150 140 130 120 110 100 90 80 70 60 50 40 30 ppm

b)

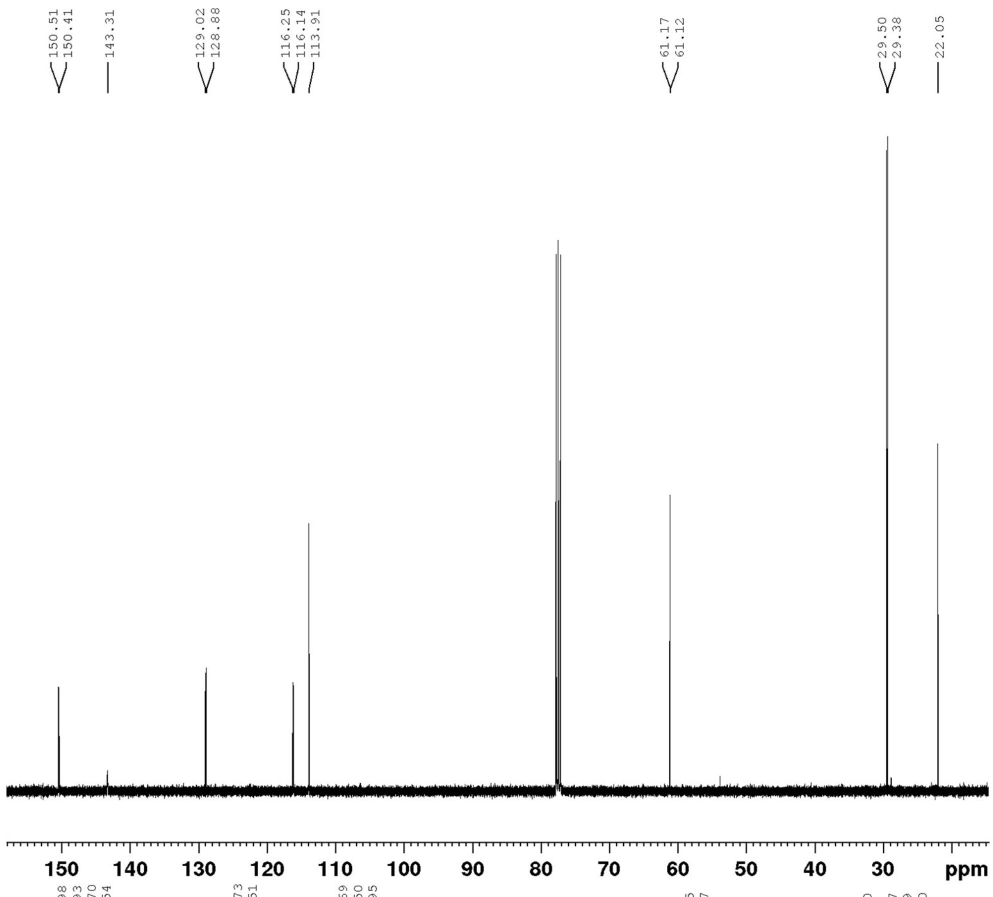
150.51
150.41
143.31
129.02
128.88
116.25
116.14
113.91
61.17
61.12
29.50
29.38
22.05
150
140
130
120
110
100
90
80
70
60
50
40
30
ppm

c)

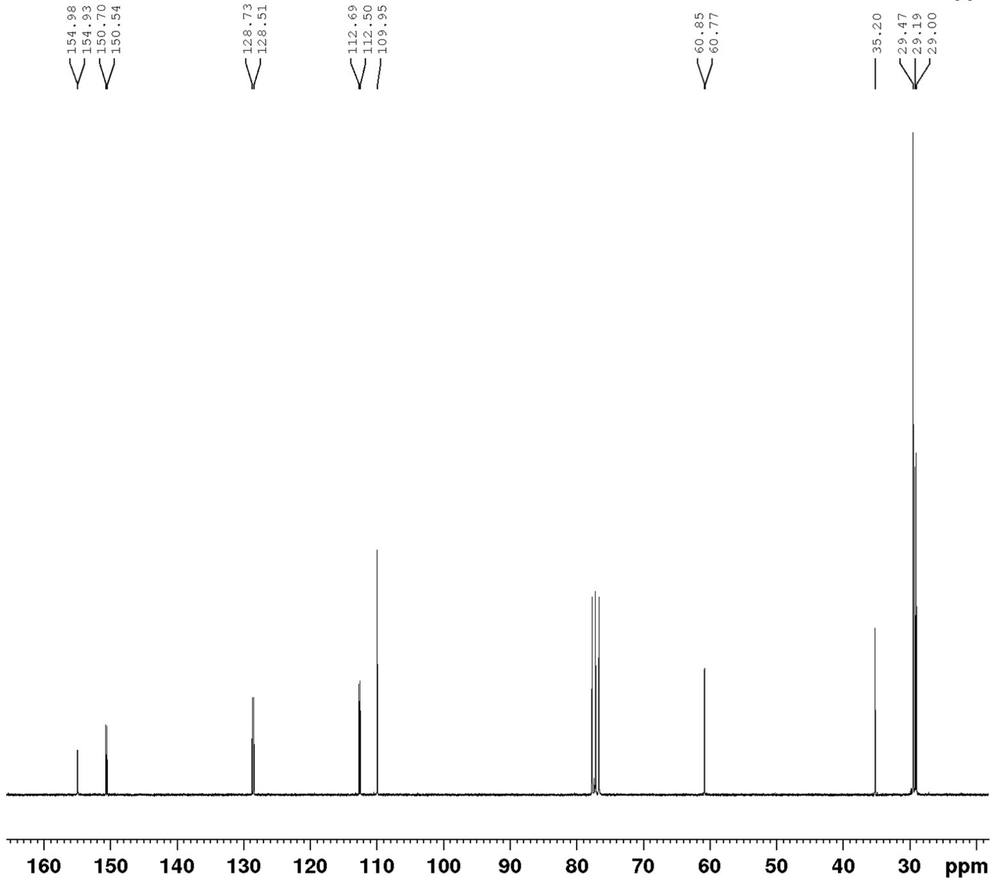
154.98
154.93
150.70
150.54
128.73
128.51
112.69
112.50
109.95
60.85
60.77
35.20
29.47
29.19
29.00
160
150
140
130
120
110
100
90
80
70
60
50
40
30
ppm

d)

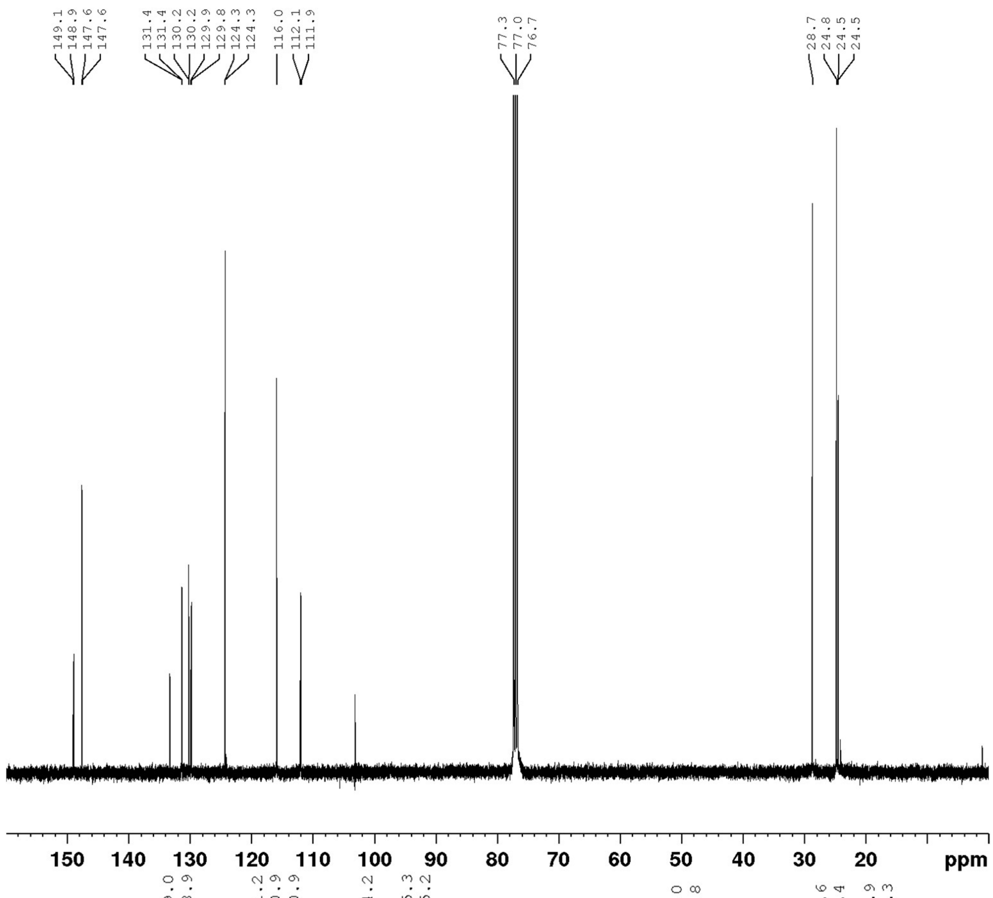
149.1
148.9
147.6
147.6
131.4
131.4
130.2
130.2
129.9
129.8
124.3
124.3
116.0
112.1
111.9
77.3
77.0
76.7
28.7
24.8
24.5
24.5
150 140 130 120 110 100 90 80 70 60 50 40 30 20 ppm

e)

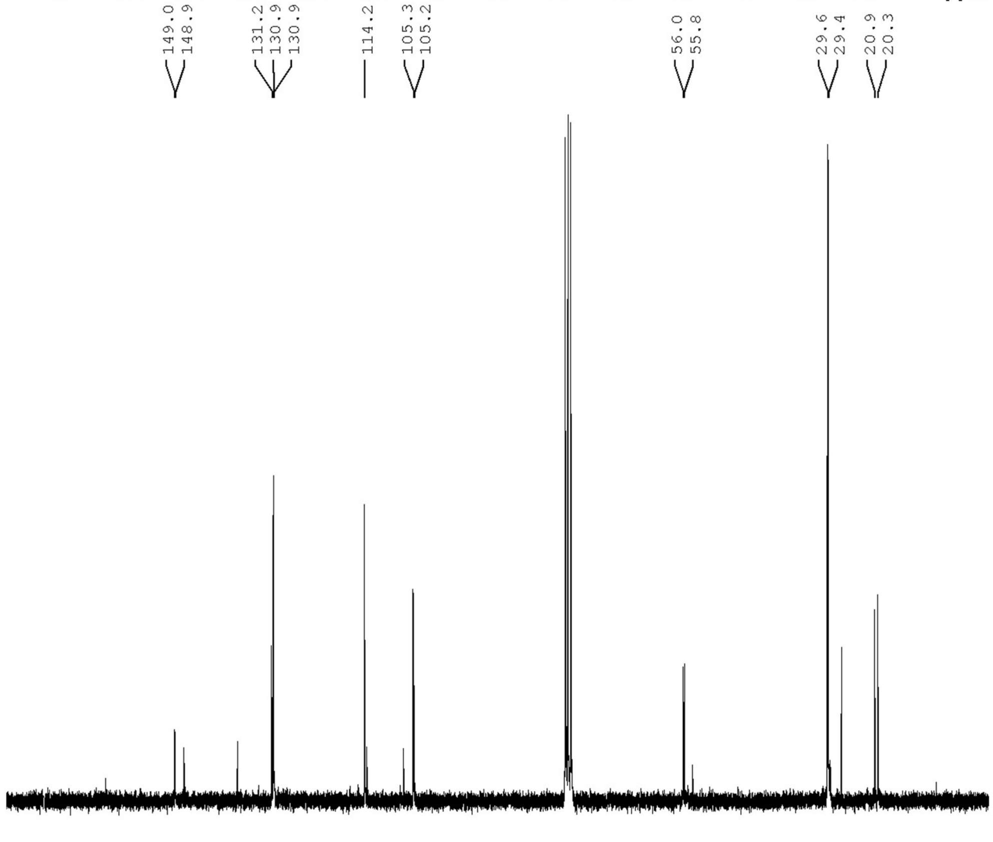
149.0
148.9
131.2
130.9
130.9
114.2
105.3
105.2
56.0
55.8
29.6
29.4
20.9
20.3
160 140 120 100 80 60 40 20 ppm

f)

g) 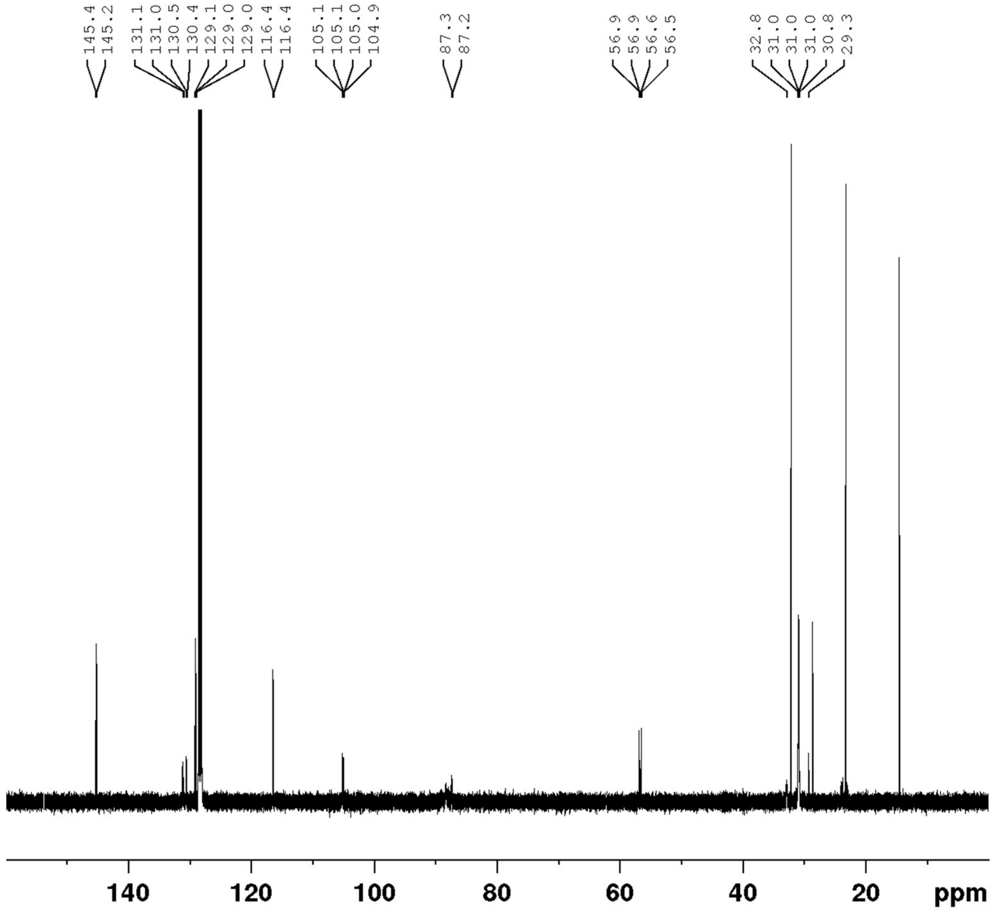

Figure S3: $^{13}C\{^{1}H\}$ NMR spectra (in $CDCl_3$) for **2b** (a), **2c** (b), **2d** (c), **2e** (d), **2f** (e), **4** (f), and **5** (g).

a) 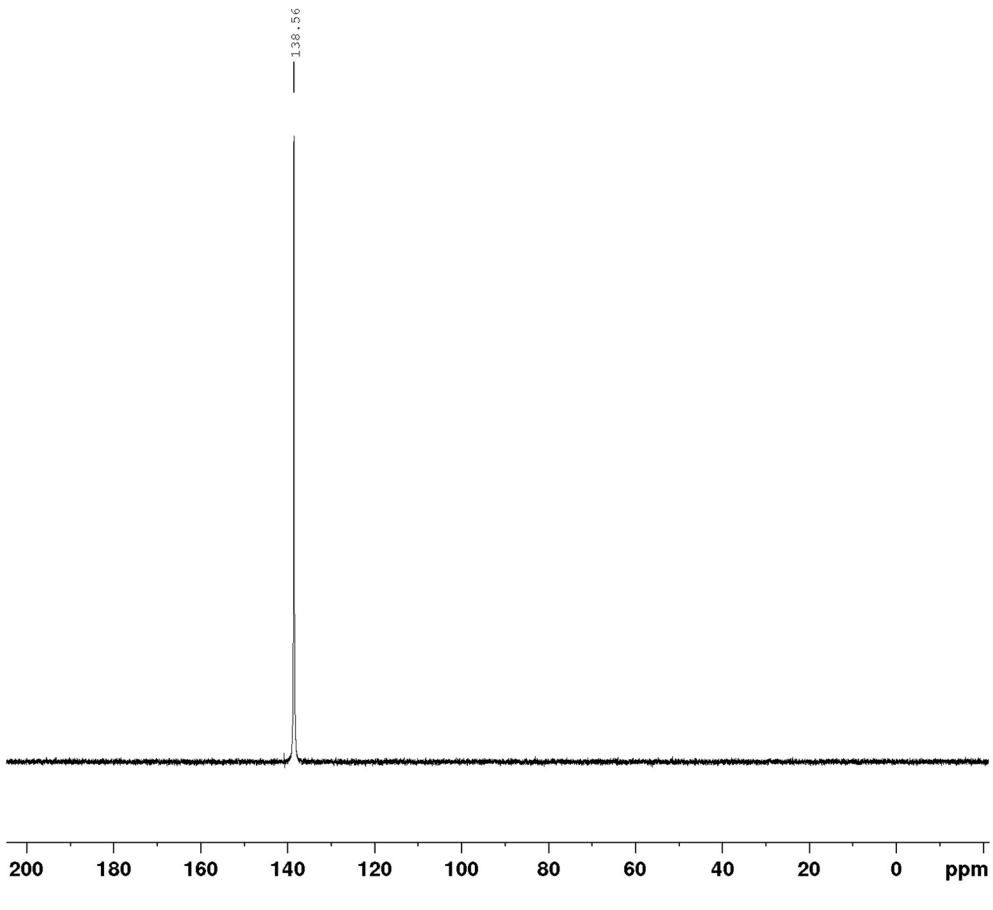

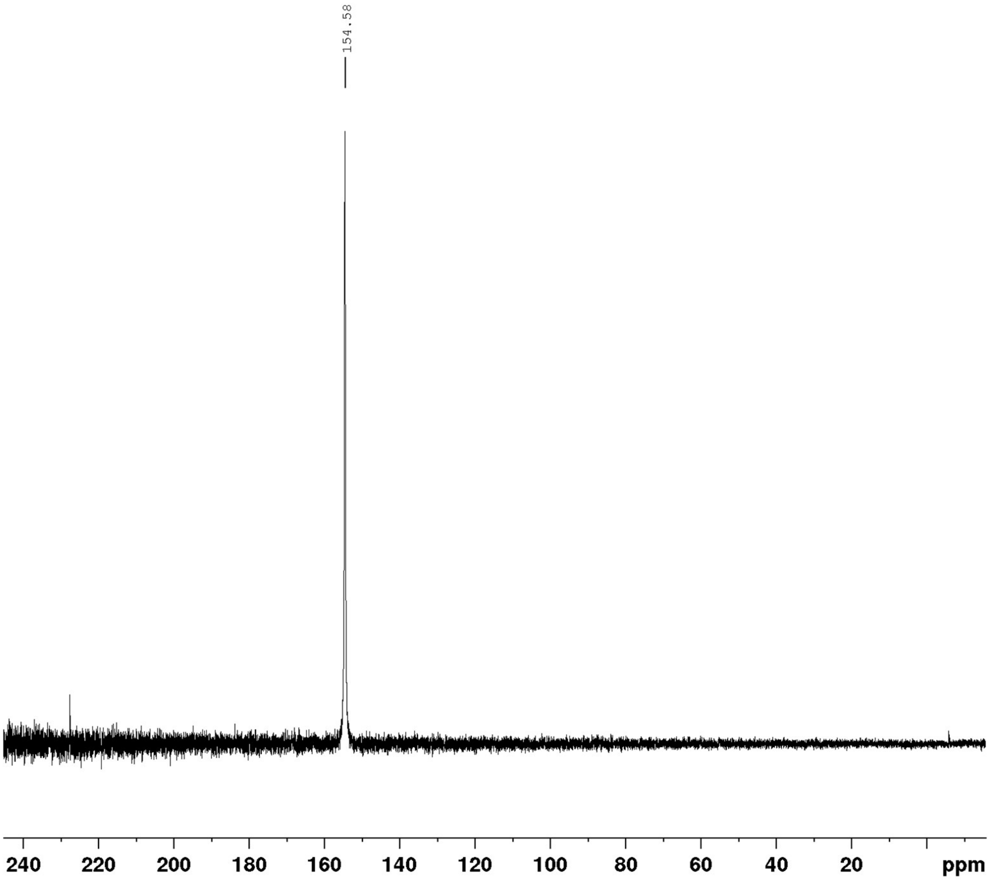
154.58
240
220
200
180
160
140
120
100
80
60
40
20
ppm
b)

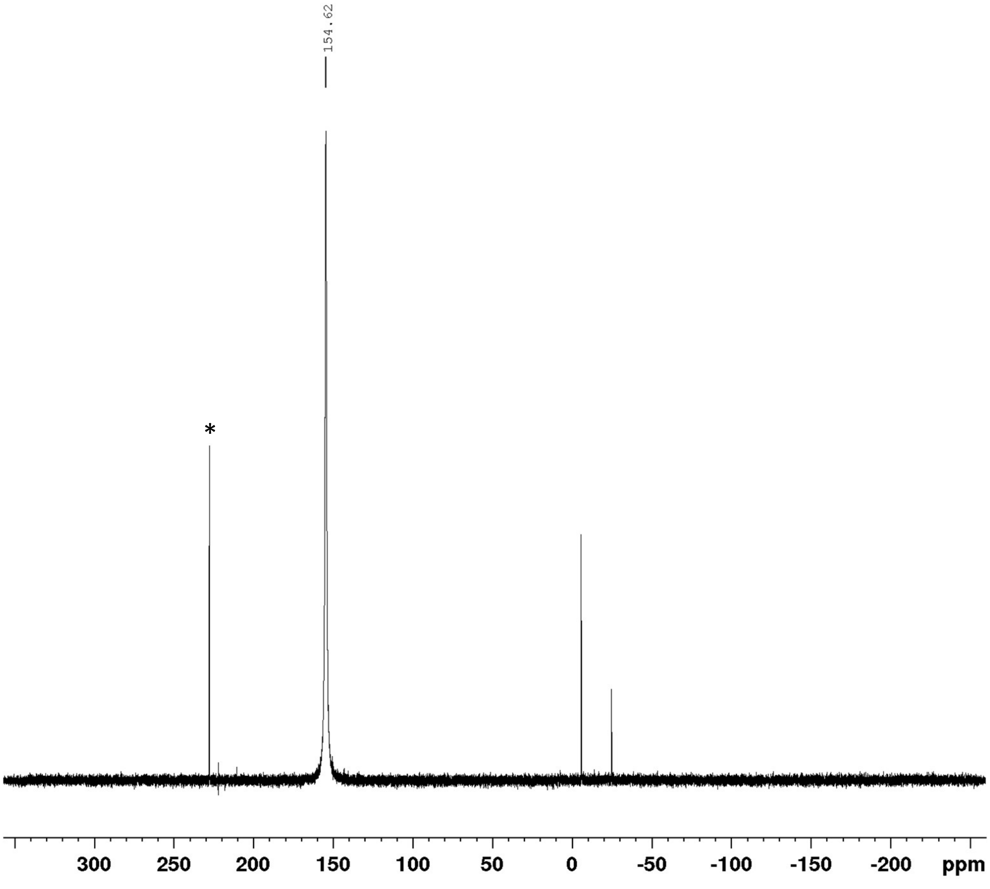
154.62
*
300
250
200
150
100
50
0
-50
-100
-150
-200
ppm
c)

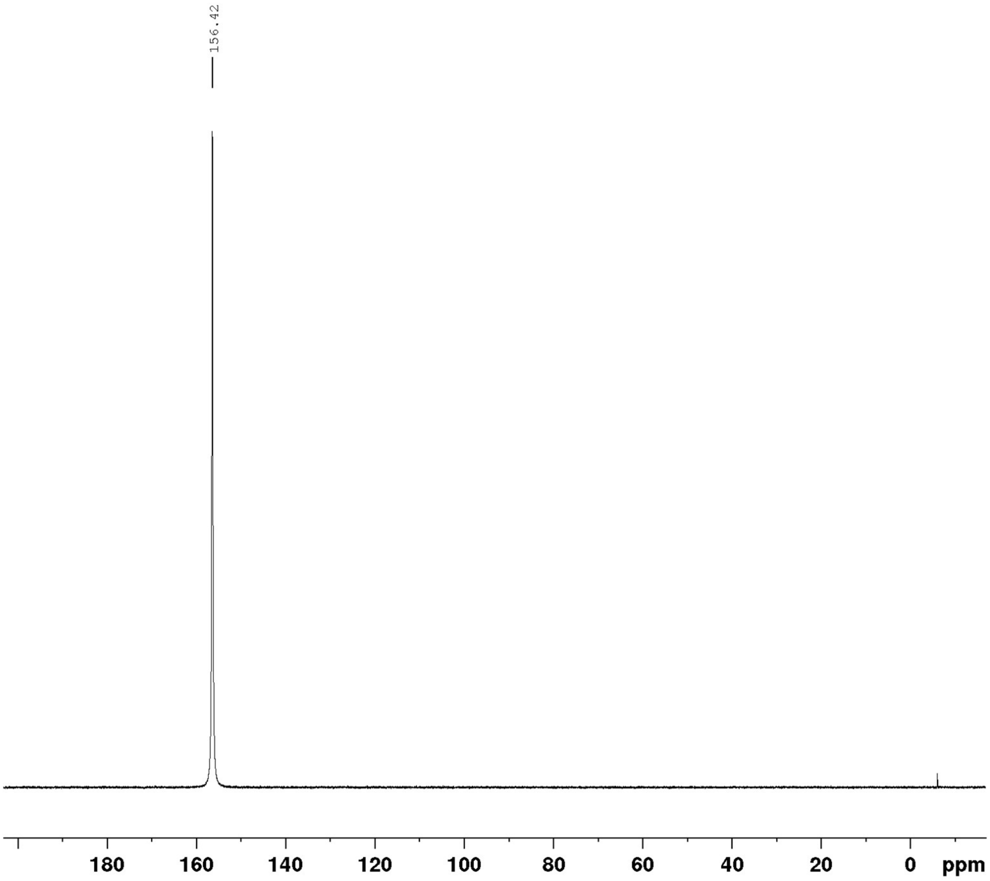
156.42
180
160
140
120
100
80
60
40
20
0
ppm

d)

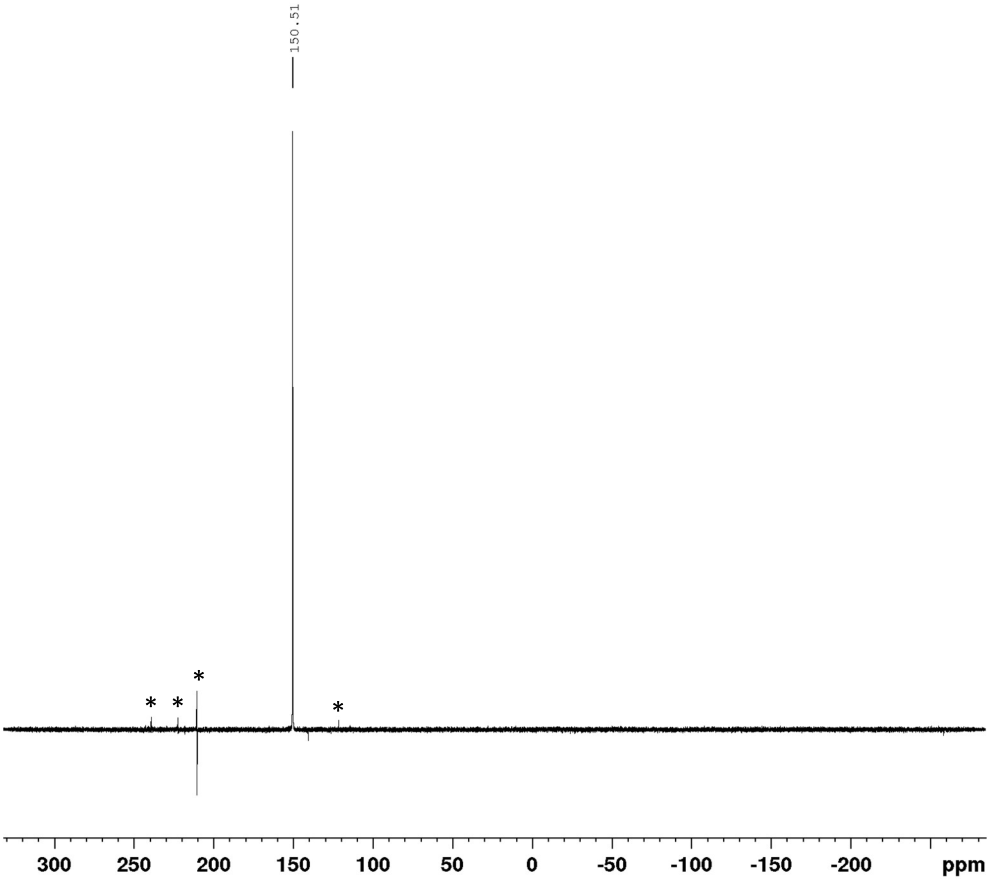
150.51
*
*
*
*
300
250
200
150
100
50
0
-50
-100
-150
-200
ppm

e)

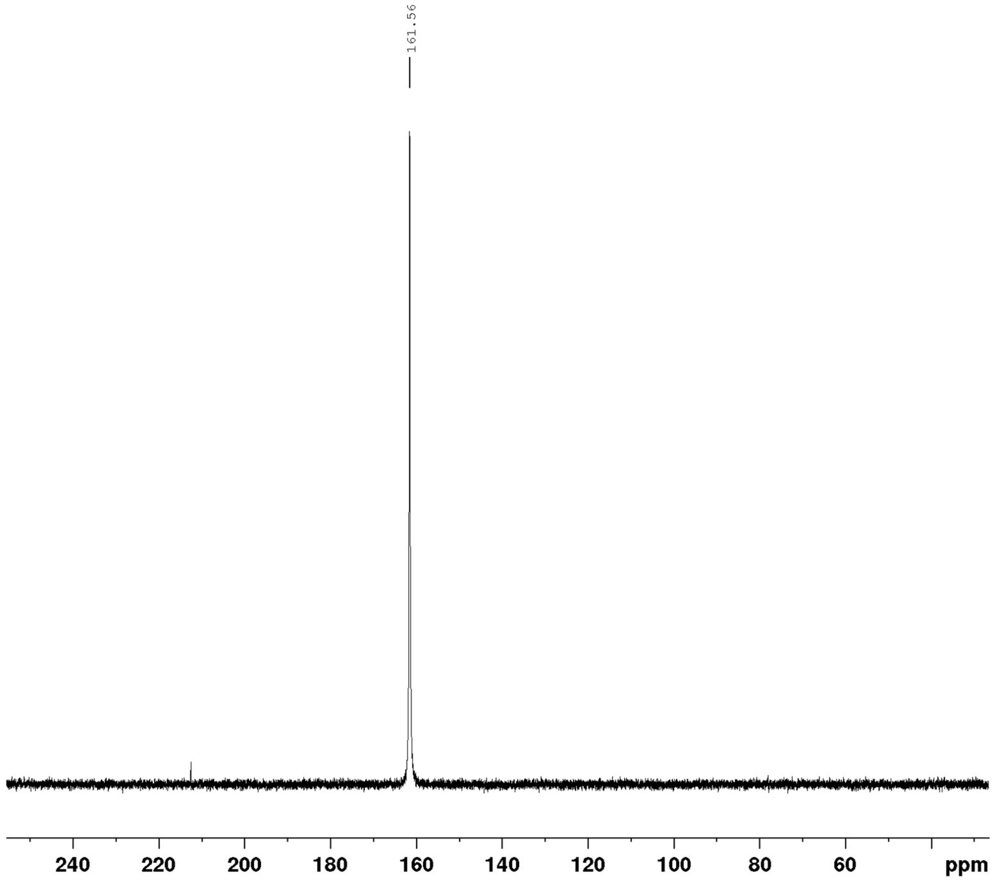
161.56
240 220 200 180 160 140 120 100 80 60 ppm

f)

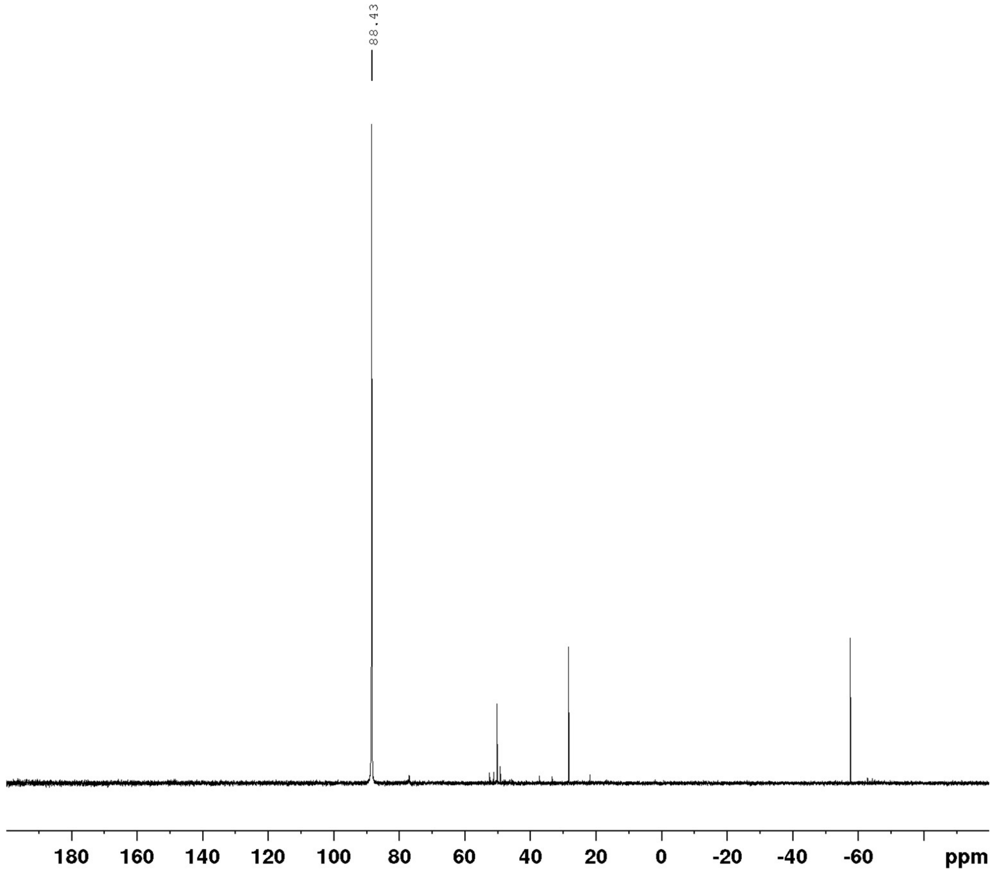
88.43
180 160 140 120 100 80 60 40 20 0 -20 -40 -60 ppm

g)

h)

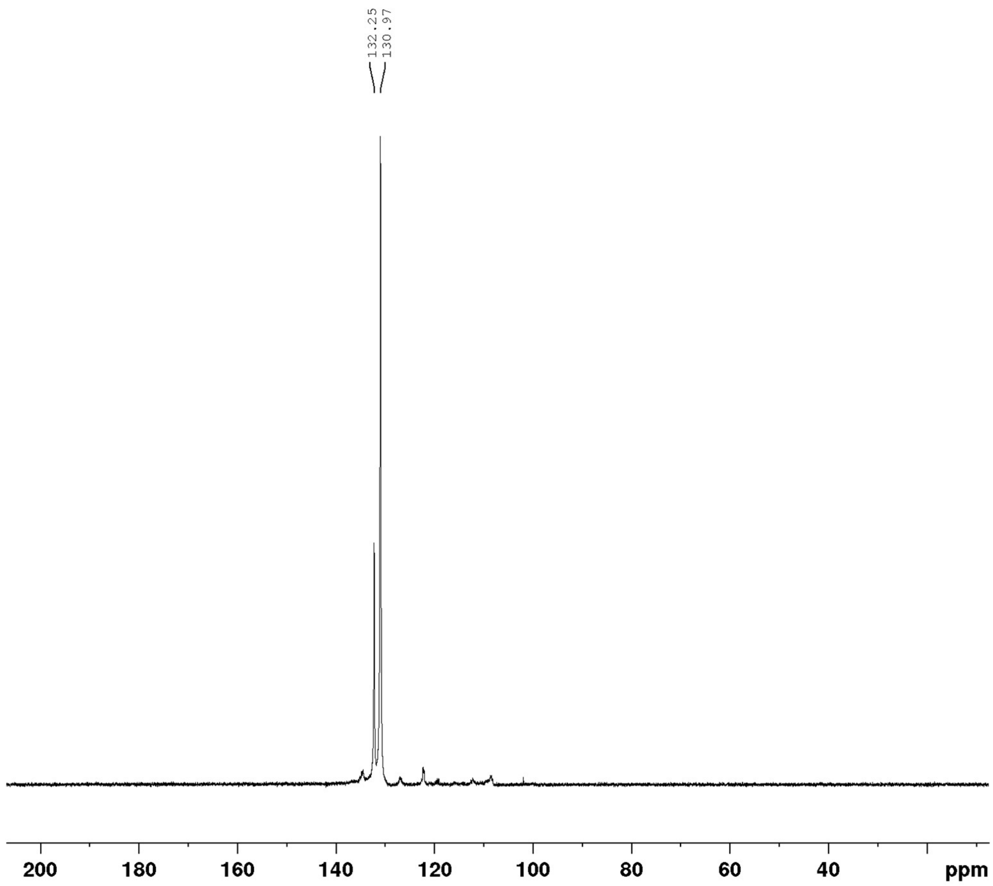

Figure S4 $^{31}P\{^1H\}$ NMR spectra (in $CDCl_3$) for **2b** (a), **2c** (b), **2d** (c), **2e** (d), **2f** (e), **3a** (f), **4** (g), and **5** (h) * denote electronic spikes
